# Long-term Outcomes of the Effects of Home Blood Pressure Telemonitoring and Pharmacist Management on Blood Pressure Among Adults With Uncontrolled Hypertension: Follow-up of a Cluster Randomized Clinical Trial

**DOI:** 10.1001/jamanetworkopen.2018.1617

**Published:** 2018-09-07

**Authors:** Karen L. Margolis, Stephen E. Asche, Steven P. Dehmer, Anna R. Bergdall, Beverly B. Green, JoAnn M. Sperl-Hillen, Rachel A. Nyboer, Pamala A. Pawloski, Michael V. Maciosek, Nicole K. Trower, Patrick J. O’Connor

**Affiliations:** 1 HealthPartners Institute for Education and Research, Minneapolis, Minnesota; 2 Kaiser Permanente Washington Health Research Institute, Seattle

## Abstract

**Importance:**

Hypertension is a leading cause of cardiovascular disease. The results were previously reported of a trial of home blood pressure (BP) telemonitoring and pharmacist management intervention in which the interventions stopped after 12 months. There were significantly greater reductions in systolic BP (SBP) in the intervention group than in the usual care group at 6, 12, and 18 months (−10.7, −9.7, and −6.6 mm Hg, respectively).

**Objectives:**

To examine the durability of the intervention effect on BP through 54 months of follow-up and to compare BP measurements performed in the research clinic and in routine clinical care.

**Design, Setting, and Participants:**

Follow-up of a cluster randomized clinical trial among 16 primary care clinics and 450 patients with uncontrolled hypertension in a large health system from March 2009 to November 2015.

**Interventions:**

A home BP telemonitoring intervention with pharmacist management or usual care.

**Main Outcomes and Measures:**

Change from baseline to 54 months in SBP and diastolic BP (DBP) measured as the mean of 3 measurements obtained at each research clinic visit.

**Results:**

Among 450 patients, 228 (mean [SD] age, 62.0 [11.7] years; 54.8% male) were randomized to the telemonitoring intervention and 222 (mean [SD] age, 60.2 [12.2] years; 55.9% male) to usual care. Research clinic BP measurements were obtained from 326 of 450 (72.4%) study patients at the 54-month follow-up visit, including 162 (mean [SD] age, 62.0 [11.1] years; 54.9% male) randomized to the telemonitoring intervention and 164 (mean [SD] age, 60.0 [11.2] years; 57.3% male) to usual care. Routine clinical care BP measurements were obtained from 439 of 450 (97.6%) study patients at 6248 visits during the follow-up period. Based on research clinic measurements, baseline mean SBP was 148 mm Hg in both groups. In the intervention group, mean SBP at 6-, 12-, 18-, and 54-month follow-up was 126.7, 125.7, 126.9, and 130.6 mm Hg, respectively. In the usual care group, mean SBP at 6-, 12-, 18-, and 54-month follow-up was 136.9, 134.8, 133.0, and 132.6 mm Hg, respectively. The differential reduction by study group in SBP from baseline to 54 months was −2.5 mm Hg (95% CI, −6.3 to 1.2 mm Hg; *P* = .18). The DBP followed a similar pattern, with a differential reduction by study group from baseline to 54 months of −1.0 mm Hg (95% CI, −3.2 to 1.2 mm Hg; *P* = .37). The SBP and DBP results from routine clinical measurements suggested significantly lower BP in the intervention group for up to 24 months.

**Conclusions and Relevance:**

This intensive intervention had sustained effects for up to 24 months (12 months after the intervention ended). Long-term maintenance of BP control is likely to require continued monitoring and resumption of the intervention if BP increases.

**Trial Registration:**

ClinicalTrials.gov Identifier: NCT00781365

## Key Points

**Question:**

How long does blood pressure remain lower compared with usual care after a 12-month intensive intervention (home telemonitoring and pharmacist management)?

**Findings:**

In this follow-up of a cluster randomized trial of 326 patients with uncontrolled hypertension, research clinic measurements showed that home blood pressure telemonitoring with pharmacist management lowered blood pressure more than usual care in the first 18 months, but this was not sustained through 54 months. The results from routine clinical measurements suggested significantly lower blood pressure in the intervention group for up to 24 months.

**Meaning:**

Long-term maintenance strategies may be needed to sustain blood pressure intervention effects over several years.

## Teaser

This follow-up of a cluster randomized clinical trial of adults with uncontrolled hypertension examines the durability of the effect of a home blood pressure telemonitoring and pharmacist management intervention on blood pressure through 54 months of follow-up and compares blood pressure measurements performed in the research clinic vs usual care.

## Introduction

Hypertension affects about 30% of US adults, has estimated costs exceeding $50 billion annually, and is the most common chronic condition for which patients see primary care physicians.^1,2^ Decades of research with various well-tolerated, effective, and inexpensive drugs have shown that treatment of hypertension prevents cardiovascular events.^3,4,5,6^ Although blood pressure (BP) control has improved over the past 2 decades, it is controlled to recommended levels in only about one-half of American adults with hypertension.^2,7^

Studies^8,9,10,11,12,13,14^ conducted in a variety of settings have found that telemedicine interventions can significantly improve hypertension management when combined with nurse-led or pharmacist-led care. The results were previously reported of a cluster randomized clinical trial evaluating home BP telemonitoring with pharmacist management compared with usual care (UC), with significant reductions in BP favoring the intervention group over 18 months.^15^ However, a barrier to implementing similar interventions in clinical practice is the scarcity of data on long-term treatment results beyond 12 months.^16,17^

To better understand the effect of telemedicine on long-term hypertension outcomes, the present analysis examined the durability of the intervention effect on BP through 54 months of follow-up in the 2 study treatment groups (telemonitoring intervention [TI] and UC). We also examined the patterns of BP by treatment group measured in routine clinical care as recorded in the electronic health record (EHR) to determine if similar patterns were observed between research clinic BP and routine care BP measurements.

## Methods

### Design, Setting, and Patients

**Figure 1.**
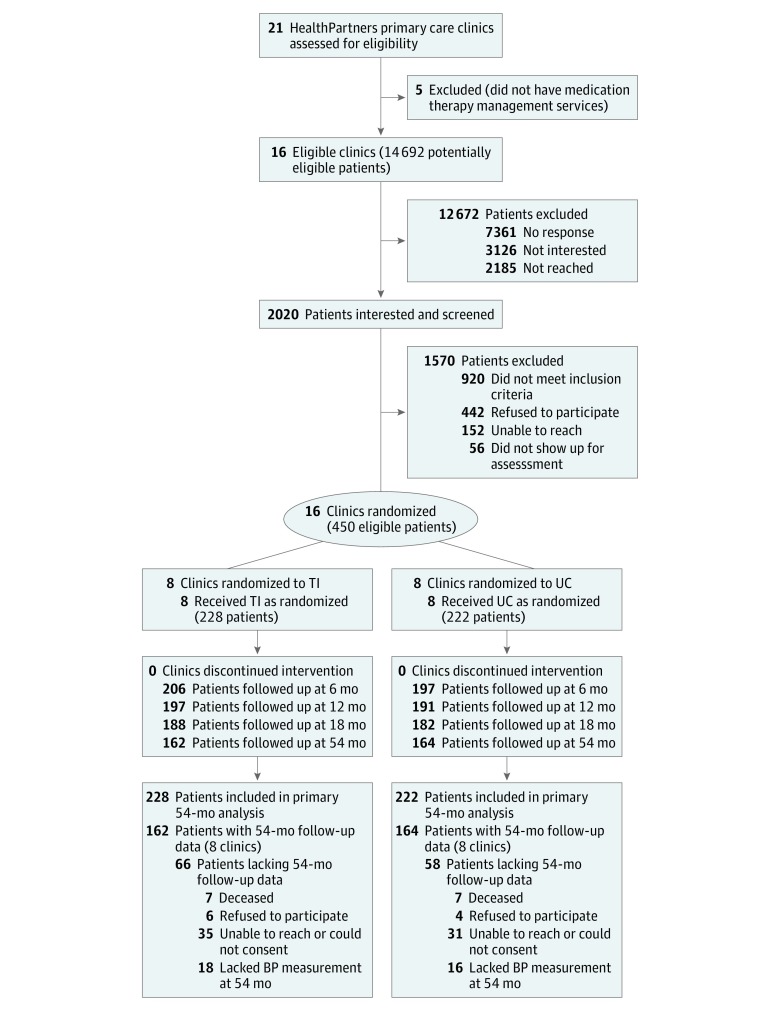
Participant Recruitment, Enrollment, and Follow-up BP indicates blood pressure; TI, telemonitoring intervention; and UC, usual care.

This 2-group follow-up of a cluster randomized clinical trial was conducted from March 2009 to November 2015 among 16 primary care clinics and 450 patients with uncontrolled hypertension at HealthPartners Medical Group, a multispecialty practice in the Minneapolis–St Paul metropolitan area of Minnesota that is part of an integrated health system. The study design and methods are described in detail in the Supplement and in previous reports,^15,18,19,20,21,22^ and recruitment, enrollment, and follow-up of the study cohort are shown in Figure 1. Briefly, we used electronic data to identify individuals and mail recruitment letters between March 2009 and April 2011 to 14 492 adult patients who had BP of 140/90 mm Hg or higher at the 2 most recent primary care encounters in the previous year. Medical exclusion criteria included the following: stage 4 or 5 kidney disease or albumin to creatinine ratio exceeding 700 mg per gram of creatinine; acute coronary syndrome, coronary revascularization, or stroke within the past 3 months; known secondary causes of hypertension; pregnancy; class III or IV New York Heart Association heart failure; or known left ventricular ejection fraction less than 30%.

Study participants were further required to have uncontrolled BP (≥140/90 mm Hg or ≥130/80 mm Hg if diabetes or kidney disease was present) based on the mean of 3 automated measurements taken using a standardized protocol in the research clinic. Of 2020 patients who were screened for the study, 450 were eligible and agreed to participate. Among 21 HealthPartners primary care clinics, 16 had a medication therapy management (MTM) pharmacist on site in 2009 at least once weekly.^23^ At these clinics, there was a collaborative practice agreement between pharmacists and primary care physicians that allowed pharmacists to prescribe and change antihypertensive therapy according to a specified protocol. The 16 study clinics were randomly assigned to either the TI group (8 clinics with 228 patients) or the UC group (8 clinics with 222 patients). Patients were assigned a treatment based on the location of their primary care physician in a TI or UC clinic.

All enrolled patients provided written informed consent. All consenting patients were masked to treatment assignment before randomization, but treatment was necessarily unmasked after randomization. The study protocol was approved by the HealthPartners institutional review board. This study and the prior study followed Consolidated Standards of Reporting Trials (CONSORT) reporting guidelines.

### Interventions

The patients in the TI group received a home automated oscillometric BP monitor (767PC; A&D Medical) that stored and transmitted BP data to a secure website (AMC Health). Pharmacists met with patients in person for a 1-hour intake visit during which they conducted a personalized medication review, taught them how to use the home BP telemonitoring system, and provided information about hypertension management. Patients were instructed to transmit at least 6 BP measurements weekly (3 in the morning and 3 in the evening) and were given an individualized home BP goal (ie, <135/85 mm Hg or <125/75 mm Hg for patients with diabetes or kidney disease). During the first 6 months of the intervention, patients and pharmacists met every 2 weeks via telephone until BP control was sustained for 6 weeks, and then the frequency was reduced to monthly. During the second 6 months of the intervention period, telephone visits were reduced to every 2 months. After 12 months, patients returned the telemonitors, went back to their primary physicians’ care, and received no further pharmacist support unless they or their physicians sought it outside of the study.

Telephone visits included review of home BP data, discussion of adherence to medication and lifestyle changes, and treatment issues that patients might be experiencing. Pharmacists were asked to adjust antihypertensive drug therapy if less than 75% of readings since the last visit met the BP goal. Regardless of BP control, the drug dosage could be lowered or the drug changed if patients experienced adverse effects. Pharmacists communicated with patients’ primary care teams through the EHR after each visit.

During the study period, patients in the UC group worked with their primary care physicians as usual. This could include referral to an MTM pharmacist for consultation (1-2 visits without telephone follow-up or prolonged monitoring) and conventional home BP measurement.

### Outcomes

This long-term assessment was planned after the study results through 18 months were analyzed. The primary outcome was change in systolic BP (SBP) from baseline to the 54-month research clinic visit. The BP and other outcomes at 6, 12, and 18 months are reported herein for context but were previously published.^15^ Other outcomes included changes from baseline to 54 months in diastolic BP (DBP) and the number of antihypertensive medication classes recorded from the medication inventory at each visit. Outcomes from patient surveys included 1 item concerning use of a home BP monitor, 3 items assessing self-efficacy for managing BP,^10^ and 2 items recording the addition of salt when cooking or at the table. Demographic data were collected at baseline and included sex, race/ethnicity, education level, marital status, and household income. Blood pressure was measured in all participants at research clinic visits at baseline and 6, 12, 18, and 54 months using a standardized technique. Three BP measurements were taken at 1-minute intervals at each research clinic visit. They were performed by a trained coordinator (R.A.N. and 2 nonauthors) who manually triggered an automated BP monitor identical to the model used for home BP monitoring. For the 54-month BPs, the BP was based on the mean of 6 BPs from 2 visits for patients having both visits (n = 282) or on the mean of 3 BPs from 1 visit for patients having 1 visit (n = 44). For all other time points, 3 BPs at a single visit were averaged.

### Sample Size and Statistical Analysis

Based on research clinic visits in the main study through 18 months, it was expected that 65% to 80% of the original 450 study enrollees would complete a 54-month visit. Assuming a 54-month visit completion rate of 70%, 16 clinics (clusters), equal cluster size, and a clinic-level intraclass correlation coefficient (ICC) for SBP in the observed range of the main study at 0.01, this study was powered at 80% to detect a difference in SBP between the TI group and the UC group at 54 months of 5.2 mm Hg (α = .05, 2-sided test).

For the primary outcome of change in SBP and for other continuously distributed outcomes, we used general linear mixed models predicting SBP or DBP from treatment group, time, and the treatment group by time interaction and included a random intercept for clinic to accommodate clinic randomization and covariances among residuals to accommodate repeated measurements from patients. The analysis used all available BP data at baseline (n = 450) and all later time points. This approach followed the modeling steps by Diggle et al^24^ in which an overelaborate means model is estimated, random effects are selected for the covariance model, the covariance matrix for residuals is selected, and tests for fixed effects are conducted. The best-fitting model for both SBP and DBP used unstructured means and an unstructured covariance matrix. Contrasts were used to estimate differential change in BP across treatment groups from baseline to 54 months. Using such direct likelihood-based ignorable methods yields valid inference when outcome data are missing at random.^25,26^ The analysis of binary outcomes used generalized linear mixed models with a logit link and treatment group, time, the treatment group by time interaction, and a random intercept for clinic.

In sensitivity analyses for the analysis of SBP and DBP, the models were set up as a postintervention analysis of BP at 54 months and conditioned on baseline BP and factors predictive of missing visits at 54 months. These models included the number of antihypertensive medication classes at baseline, education level (some college vs high school or less), marital status (married or cohabiting vs other), and work status (full-time vs other). Household income also predicted missing visits at 54 months but was often missing and thus not used as a covariate in the model.

The BP patterns were also summarized using BP data gathered from the EHR at clinic visits not associated with the research clinic visits. These data were used to compare BP measurements performed in the research clinic and in routine clinical care and to understand the BP trajectory during the period 18 to 54 months after the baseline visit because no research clinic visits occurred during this time. All EHR BPs were gathered from 6248 visits made by 439 of the 450 (97.6%) original study enrollees at departments of family practice, internal medicine, obstetrics/gynecology, geriatrics, nursing, diabetes education program, MTM, cardiology, and endocrinology. We predicted the mean BP and 95% CI using a polynomial random coefficients model with a random intercept and slope for patients; terms for linear, quadratic, and cubic time; treatment group; and interactions of treatment group and time terms. Analysis was conducted with statistical software (SAS, version 9.4; SAS Institute Inc).

## Results

Among 450 patients, 228 (mean [SD] age, 62.0 [11.7] years; 54.8% male) were randomized to the TI and 222 (mean [SD] age, 60.2 [12.2] years; 55.9% male) to UC. Among the patients in the original sample who completed a baseline clinic visit, 31 were never reachable after baseline (n = 24) or died (n = 7) in the original study phase, leaving 419 eligible for the extended follow-up. Among those eligible, an additional 7 were deceased, 10 declined, 3 could not consent because of dementia or nursing home placement, and 39 were not responsive to the study invitations during the extended follow-up phase. The profile of reasons for nonresponse was similar by treatment group (Figure 1). Among the remaining 360, a total of 326 (72.4% of the original 450 patients; 162 TI and 164 UC) had the first 54-month study visit, at which their BP was measured (296 at the research clinic and 30 by a research coordinator [R.A.N.] at the patient’s home or primary care clinic), and 282 patients (135 TI and 147 UC) returned 2 weeks later to have their BP measured at the second follow-up visit. The overall study follow-up rates for the first 54-month visit were 71.1% (162 of 228; mean [SD] age, 62.0 [11.1] years; 54.9% male) in the TI and 73.9% (164 of 222; mean [SD] age, 60.0 [11.2] years; 57.3% male) in UC (*P* = .50) (Figure 1). The mean (SD) duration between the baseline and first 54-month visit was 54.3 (0.7) months. The mean (SD) duration between the first and second 54-month visits was 2.3 (0.7) weeks.

Baseline characteristics of patients by study group are listed in Table 1 for the original study sample (n = 450) and the sample with a study-measured BP at 54 months (n = 326). Patients at baseline had a mean (SD) age of 61.1 (12.0) years, 249 (55.3%) were male, and 368 (81.7%) were of white race/ethnicity. About half (47.9% [209 of 436]) had earned a college degree, and 40.5% (176 of 435) were working full-time. The mean BP was 148/85 mm Hg at baseline. Hispanic participants made up a larger proportion of the UC group than the TI group in the baseline sample. There were no other statistically significant differences in patient characteristics by study group in either the baseline or follow-up samples. Compared with patients who did not complete the 54-month visit (n = 124), patients who completed the 54-month visit (n = 326) had fewer antihypertensive medication classes at baseline (mean, 1.5 classes for completers vs 1.9 classes for noncompleters; *P* < .001), were more educated (86.2% [275 of 319] had some college compared with 72.7% [85 of 117]; *P* < .001), were more likely to be married or cohabiting at baseline (73.6% [234 of 318] vs 57.3% [67 of 117]; *P* = .001), were more likely to be working full-time (43.6% [139 of 319] vs 31.9% [37 of 316]; *P* = .03), and had a higher household income (70.8% [199 of 281] of completers had household incomes exceeding $50 000 compared with 54.5% [55 of 101] of noncompleters; *P* = .003).

**Table 1.** Baseline Characteristics of All Patients and Patients Completing the 54-Month Visit

Variable	All Patients (n = 450)		Patients Completing 54-mo Visit (n = 326)	
	TI (n = 228)	UC (n = 222)	TI (n = 162)	UC (n = 164)
Male, No. (%)	125 (54.8)	124 (55.9)	89 (54.9)	94 (57.3)
Age, mean (SD), y	62.0 (11.7)	60.2 (12.2)	62.0 (11.1)	60.0 (11.2)
Race/ethnicity self-reported by participants, No. (%)^a^				
White	191 (83.8)	177 (79.7)	140 (86.4)	135 (82.3)
Black	24 (10.5)	29 (13.1)	15 (9.3)	18 (11.0)
Asian	4 (1.8)	3 (1.4)	2 (1.2)	3 (1.8)
Other	9 (4.0)	13 (5.9)	5 (3.1)	8 (4.9)
Hispanic^b^	1 (0.4)	9 (4.1)	1 (0.6)	5 (3.1)
Diabetes, No. (%)	46 (20.2)	40 (18.0)	29 (17.9)	33 (20.1)
History of CVD, No. (%)	23 (10.1)	20 (9.0)	14 (8.6)	15 (9.2)
Taking any BP medication, No. (%)	176 (77.2)	159 (71.6)	125 (77.2)	114 (69.5)
No. of BP medication classes, mean (SD)^c^	1.7 (1.3)	1.5 (1.2)	1.6 (1.3)	1.3 (1.2)
Education level, No./total No. (%)^c^				
High school or less	36/221 (16.3)	40/215 (18.6)	21/158 (13.3)	23/161 (14.3)
Some college	72/221 (32.6)	79/215 (36.7)	53/158 (33.5)	62/161 (38.5)
4-y Degree	46/221 (20.8)	36/215 (16.7)	39/158 (24.7)	29/161 (18.0)
>4-y Degree	67/221 (30.3)	60/215 (27.9)	45/158 (28.5)	47/161 (29.2)
Married or cohabiting, No./total No. (%)^c^	160/221 (72.4)	141/214 (65.9)	121/158 (76.6)	113/160 (70.6)
Work status, No./total No. (%)^c^				
Full-time	86/221 (38.9)	90/214 (42.1)	67/158 (42.4)	72/161 (44.7)
Part-time	28/221 (12.7)	25/214 (11.7)	17/158 (10.8)	16/161 (9.9)
Retired	87/221 (39.4)	76/214 (35.5)	63/158 (39.9)	59/161 (36.7)
Not working	20/221 (9.1)	23/214 (10.8)	11/158 (7.0)	14/161 (8.7)
Household income, $, No./total No. (%)^c^				
<30 000	34/187 (18.2)	31/195 (15.9)	17/132 (12.9)	17/149 (11.4)
30 000-49 999	27/187 (14.4)	36/195 (18.5)	22/132 (16.7)	26/149 (17.5)
50 000-99 999	69/187 (36.9)	81/195 (41.5)	45/132 (34.1)	71/149 (47.7)
≥100 000	57/187 (30.5)	47/195 (24.1)	48/132 (36.4)	35/149 (23.5)
BP, mean (SD), mm Hg				
Baseline systolic	148.2 (12.9)	147.7 (13.2)	147.8 (12.3)	147.1 (12.2)
Baseline diastolic	84.5 (11.7)	84.9 (11.5)	84.1 (11.6)	85.3 (10.9)

Abbreviations: BP, blood pressure; CVD, cardiovascular disease; TI, telemonitoring intervention; UC, usual care.

^a^ Percentages sum to greater than 100% because some participants self-identified as more than 1 race/ethnicity.

^b^ *P* < .05. Patient characteristic differs by study group in the baseline sample.

^c^ Patient characteristic in completers of the 54-month follow-up differs from noncompleters.

As previously reported,^15^ the mean SBP was reduced from 148 mm Hg in both groups to 126.7, 125.7, 126.9, and 130.6 mm Hg at 6-, 12-, 18-, and 54-month follow-up in the TI group and 136.9, 134.8, 133.0, and 132.6 mm Hg at 6-, 12-, 18-, and 54-month follow-up in the UC group. The mean differences in SBP change between the TI group and the UC group were −10.7 mm Hg (95% CI, −14.3 to −7.3 mm Hg) from baseline to 6 months (*P* < .001), −9.7 mm Hg (95% CI, −13.4 to −6.0 mm Hg) from baseline to 12 months (*P* < .001), and −6.6 mm Hg (95% CI, −10.7 to −2.5 mm Hg) from baseline to 18 months (*P* = .004) (Table 2). Data from the long-term follow-up indicate that the mean SBP was 130.6 mm Hg at 54 months for patients in the TI group (reduction of −17.6 mm Hg from baseline; *P* < .001). For patients in the UC group, the mean SBP at 54 months was 132.6 mm Hg (reduction of −15.1 mm Hg from baseline; *P* < .001). The differential reduction by study group in SBP from baseline to 54 months was −2.5 mm Hg (95% CI, −6.3 to 1.2; *P* = .18). The clinic-level ICC for SBP at 54 months was 0.

**Table 2.** Reduction of BP From Baseline^a^

Variable	BP in TI Group, mm Hg		BP in UC Group, mm Hg		Differential Change From Baseline, mm Hg	*P* Value^b^
	Mean (95% CI)	Reduction From Baseline, Mean (95% CI)	Mean (95% CI)	Reduction From Baseline, Mean (95% CI)		
**Systolic BP**						
Baseline	148.2 (146.3 to 150.0)	NA	147.7 (145.8 to 149.5)	NA	NA	NA
6 mo	126.7 (124.4 to 129.0)	−21.5 (−23.9 to −19.1)	136.9 (134.6 to 139.2)	−10.8 (−13.3 to −8.3)	−10.7 (−14.3 to −7.3)	<.001
12 mo	125.7 (123.4 to 128.0)	−22.5 (−25.1 to −19.9)	134.8 (132.5 to 137.2)	−12.9 (−15.5 to −10.2)	−9.7 (−13.4 to −6.0)	<.001
18 mo	126.9 (124.3 to 129.4)	−21.3 (−24.2 to −18.4)	133.0 (130.4 to 135.5)	−14.7 (−17.6 to −11.8)	−6.6 (−10.7 to −2.5)	.004
54 mo	130.6 (128.2 to 133.0)	−17.6 (−20.3 to −15.0)	132.6 (130.2 to 134.9)	−15.1 (−17.7 to −12.5)	−2.5 (−6.3 to 1.2)	.18
**Diastolic BP**						
Baseline	84.4 (82.3 to 86.6)	NA	85.1 (82.9 to 87.3)	NA	NA	NA
6 mo	75.0 (72.9 to 77.2)	−9.4 (−11.1 to −7.6)	81.7 (79.5 to 84.0)	−3.4 (−5.2 to −1.5)	−6.0 (−8.6 to −3.4)	<.001
12 mo	75.1 (72.8 to 77.4)	−9.3 (−11.0 to −7.7)	80.8 (78.5 to 83.2)	−4.3 (−5.9 to −2.7)	−5.1 (−7.4 to −2.8)	<.001
18 mo	75.1 (73.0 to 77.2)	−9.3 (−11.7 to −7.0)	78.7 (76.6 to 80.9)	−6.4 (−8.7 to −3.9)	−3.0 (−6.3 to 0.3)	.07
54 mo	77.5 (75.6 to 79.4)	−7.0 (−8.5 to −5.4)	79.1 (77.2 to 81.0)	−6.0 (−7.5 to −4.4)	−1.0 (−3.2 to 1.2)	.37

Abbreviations: BP, blood pressure; NA, not applicable; TI, telemonitoring intervention; UC, usual care.

^a^ Blood pressure results from baseline and 6, 12, and 18 months were previously reported in the study by Margolis et al.^15^

^b^ Calculated using the treatment group by time interaction term, indicating a differential reduction from baseline by study group.

**Figure 2.**
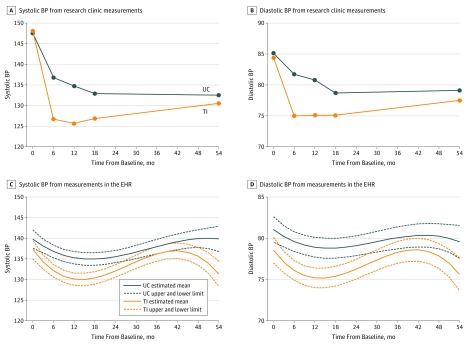
Systolic and Diastolic Blood Pressure (BP) During 54 Months of Follow-up A-D, Blood pressure in the telemonitoring intervention (TI) group and in the usual care (UC) group. C and D, Estimated mean BPs and upper and lower limits of the 95% CIs. EHR indicates electronic health record.

The main trial previously reported that the mean DBP was reduced from 84 or 85 mm Hg to 75 mm Hg in the TI group and to 81 mm Hg in the UC group during the 12-month intervention.^15^ In the present study, the mean differences in DBP change between the TI group and the UC group were −6.0 mm Hg (95% CI, −8.6 to −3.4 mm Hg) from baseline to 6 months (*P* < .001), −5.1 mm Hg (95% CI, −7.4 to −2.8 mm Hg) from baseline to 12 months (*P* < .001), and −3.0 mm Hg (95% CI, −6.3 to 0.3 mm Hg) from baseline to 18 months (*P* = .07). Data from the long-term follow-up indicate that the mean DBP was 77.5 mm Hg at 54 months for patients in the TI group (reduction of −7.0 mm Hg from baseline; *P* < .001). For patients in the UC group, the mean DBP at 54 months was 79.1 mm Hg (reduction of −6.0 mm Hg from baseline; *P* < .001). The differential reduction by study group in DBP from baseline to 54 months was −1.0 mm Hg (95% CI, −3.2 to 1.2; *P* = .37). The clinic-level ICC for DBP at 54 months was 0.01. Most of the narrowing of study group differences in BP from 18 to 54 months was due to an increase in SBP and DBP in the TI group rather than decreases in the UC group (Figure 2A and B).

The sensitivity analyses predicting BP at 54 months from baseline BP and factors predicting missing BP data at 54 months (including the number of antihypertensive medication classes, education level, marital status, and work status) yielded information similar to the main analysis on BP reduction from baseline to 54 months. Study group differences (BP for TI minus UC) were −2.0 (95% CI, −5.8 to 1.8; *P* = .27) for SBP and −1.3 (95% CI, −3.8 to 1.2; *P* = .27) for DBP.

In total, 6248 visits with a BP measured from 439 (97.6%) of the original 450 study enrollees were gathered from the EHR for the period starting from the date of the research clinic baseline visit through 54 months after the baseline visit. The 439 patients had between 1 and 106 visits at which a BP was measured and had a median of 12 (interquartile range, 7-19) BP visits per patient. Plots of the means and 95% CIs of EHR BPs show patterns of SBP and DBP similar to research clinic measurements at 6, 12, 18, and 54 months, although the BP nadir was not as low as in the research clinic measures (Figure 2C and D). The SBP and DBP were lower in the TI group than in the UC group from 6 to 24 months after baseline, but they increased more in the TI group than in the UC group after the end of the intervention at 12 months.

The number of antihypertensive medication classes showed differentially greater increases in the TI group than the UC group when comparing baseline with 6, 12, and 18 months (Table 3). However, increases in the number of antihypertensive medication classes from baseline to 54 months were similar by group, with increases of 0.63 (95% CI, 0.46-0.79) in the TI and 0.64 (95% CI, 0.47-0.80) in UC, reflecting no differential increase (*P* = .92). The proportion of patients using a home BP monitor increased markedly at 6 and 12 months in the TI group but did not increase in the UC group. The study did not provide a home BP monitor to replace the telemonitor when it was returned to the vendor at completion of the intervention at 12 months. There was a decline in home BP monitoring in the TI group at 18 months; by 54 months, home BP monitoring was similar in the TI group (59.6%) and the UC group (50.0%), reflecting no differential long-term increase (*P* = .78). Patients’ belief in their ability to include home BP monitoring in their weekly routine declined in both groups over time.

**Table 3.** Other Study Outcomes^a^

Variable	TI					UC				
	Baseline (n = 228)	6 mo (n = 206)	12 mo (n = 197)	18 mo (n = 188)	54 mo (n = 162)	Baseline (n = 222)	6 mo (n = 197)	12 mo (n = 191)	18 mo (n = 182)	54 mo (n = 164)
No. of BP medication classes, mean	1.7	2.4	2.4	2.3	2.3	1.5	1.6	1.7	1.7	2.1
Change from baseline, mean (95% CI)	NA	0.68 (0.55 to 0.81)^b^	0.67 (0.52 to 0.82)^b^	0.65 (0.50 to 0.79)^c^	0.63 (0.46 to 0.79)	NA	0.16 (0.03 to 0.29)	0.22 (0.07 to 0.37)	0.28 (0.13 to 0.42)	0.64 (0.47 to 0.80)
Used home BP monitor in past 12 mo at baseline and in past 6 mo otherwise, %	50.6	94.1	95.4	71.4	59.6	42.8	43.7	42.8	50.7	50.0
Change, % (95% CI)	NA	43.5 (38.7 to 46.2)^b^	44.8 (40.0 to 47.0)^b^	20.8 (11.8 to 28.5)	9.2 (−1.0 to 18.6)	NA	0.9 (−8.4 to 10.6)	0.0 (−10.3 to 10.2)	7.0 (−2.6 to 17.0)	7.2 (−2.9 to 17.2)
Can include home BP monitoring in weekly routine, mean score^d^	4.6	4.7	4.2	4.0	4.0	4.5	3.8	3.7	4.0	3.5
Change, mean (95% CI)	NA	0.16 (−0.04 to 0.37)^b^	−0.34 (−0.54 to 0.14)^c^	−0.51 (−0.72 to 0.30)	−0.60 (−0.84 to −0.36)	NA	−0.69 (−0.90 to 0.48)	−0.77 (−0.97 to 0.57)	−0.50 (−0.71 to 0.28)	−0.93 (−1.17 to −0.69)
Can follow medication regimen, mean score^d^	4.7	4.8	4.7	4.8	4.7	4.7	4.5	4.6	4.6	4.6
Change, mean (95% CI)	NA	0.05 (−0.05 to 0.15)^e^	−0.08 (−0.20 to 0.05)	0.05 (−0.06 to 0.16)	0.01 (−0.09 to 0.10)	NA	−0.15 (−0.26 to 0.04)	−0.09 (−0.21 to 0.04)	−0.07 (−0.19 to 0.04)	−0.03 (−0.13 to 0.06)
Can keep BP under control, mean score^d^	3.8	4.2	4.2	4.3	4.3	3.9	3.9	3.9	4.0	4.0
Change, mean (95% CI)	NA	0.40 (0.24 to 0.55)^c^	0.34 (0.19 to 0.50)^c^	0.47 (0.30 to 0.63)^e^	0.42 (0.24 to 0.60)	NA	0.01 (−0.15 to 0.16)	0.01 (−0.14 to 0.17)	0.15 (−0.02 to 0.32)	0.19 (0.02 to 0.37)
Add salt after served at table daily or more often, %	21.1	10.3	10.4	12.3	13.0	19.4	18.9	20.9	19.3	16.5
Change, % (95% CI)	NA	−10.8 (−14.9 to −4.4)^e^	−10.7 (−14.8 to −4.1)^e^	−8.8 (−13.5 to −1.6)	−8.1 (−13.2 to −0.3)	NA	−0.5 (−8.2 to 6.9)	1.4 (−5.8 to 10.2)	−0.2 (−7.0 to 8.7)	−3.1 (−9.2 to 5.6)
Add salt when preparing food daily or more often, %	27.3	15.3	13.4	13.8	14.8	23.3	25.4	24.6	23.3	8.2
Change, % (95% CI)	NA	−12.0 (−17.3 to −4.7)^e^	−13.9 (−18.6 to −6.7)^e^	−13.5 (−18.4 to −6.2)^e^	−12.4 (−17.9 to −0.5)	NA	2.1 (−5.5 to 11.5)	1.3 (−6.3 to 10.5)	0.0 (−8.3 to 9.4)	−5.0 (−11.3 to 3.8)

Abbreviations: BP, blood pressure; NA, not applicable; TI, telemonitoring intervention; UC, usual care.

^a^ Model-based results from general and generalized linear mixed models predicting outcome from treatment group, time, and the treatment group by time interaction. *P* values from the treatment group by time interaction indicate differential change by study group from baseline to 6 months, baseline to 12 months, baseline to 18 months, or baseline to 54 months.

^b^ *P* < .001.

^c^ *P* < .01.

^d^ Items answered on scale of 1 to 5, with 1 indicating “not confident” and 5 indicating “very confident.”^10^

^e^ *P* < .05.

## Discussion

The extended follow-up of this trial testing the effects of home BP telemonitoring with pharmacist management shows no significant difference in SBP or DBP at 54 months, primarily related to an increase in BP in the TI group. Based on research clinic measurements, there was still a significant difference in SBP at 18 months, 6 months after the end of the intervention, and BP recorded in the EHR suggests that an intervention effect may persist for up to 24 months, 12 months after the end of the intervention.

Members of our research group previously reported that the 2 significant mediators of the intervention effect were increased medication treatment intensity and increased home BP monitoring, which together accounted for about 5 mm Hg of the difference in SBP at 6 months.^20^ No differences in these mediating factors were present at 54 months owing to a decrease in the proportion of patients doing home BP monitoring in the TI group and a modest increase in the antihypertensive treatment intensity in the UC group. Although this previous analysis did not find that medication adherence, self-reported salt intake, and self-efficacy were significant mediators of the intervention effect, several of those variables were more favorable in the TI group than in the UC group during the first 18 months of the study. Those differences had also become nonsignificant by 54 months. Increasing organizational efforts to meet performance metrics related to hypertension may also have contributed to erosion of significant differences in the postintervention observation period. These included mandated use of automated oscillometric BP monitors in all primary care clinics, development of hypertension registry tools for enhanced audit and feedback, and EHR reminders to repeat elevated first BP measurements.

Few previous studies have reported long-term follow-up after a successful short-term hypertension intervention. In a study^27^ comparing 6 months of pharmacist-physician collaborative management with routine primary care for patients with uncontrolled hypertension, BP became controlled in 65% of the intervention group compared with 29% of the control group. The BP control remained higher in the intervention group (67%) compared with the control group (36%) after 18 additional months of follow-up with no intervention. The results were similar in a smaller previous study^28^ conducted by the same research group. Carter et al^29^ also evaluated the effect of continuing vs discontinuing a pharmacist management intervention for uncontrolled hypertension among patients who had achieved BP control at 6 months and found that BP control was maintained regardless of whether the intervention was continued for an additional 18 months. Green et al^30^ followed up patients using EHR data after the end of a 1-year trial comparing home BP monitoring plus pharmacist management with usual primary care. The intervention group maintained an SBP reduction that was 3.6 mm Hg lower (*P* = .02) than the UC group after 6 to 18 months of additional follow-up. In an 18-month trial comparing telephone-based health behavior promotion, medication adjustments, or the combination of both, the combined intervention group had a significantly higher proportion of participants with BP control during the study period and 18 months later.^31^ These results are congruent with our findings of a sustained difference in BP for up to 24 months based on EHR data. However, none of the studies show that BP differences are maintained beyond 12 to 18 months after the end of an intervention.

A unique contribution of the present analysis is the concurrent comparison of research clinic BP measurements and clinical BP measurements extracted from the EHR. Our findings suggest that the conclusion that the intervention was effective for up to 18 months would have been similar if we had used EHR data alone. The EHR data provide further information on the trajectory of BP during the period between 18 and 54 months in which no research measurements were available. HealthPartners clinics universally adopted automated oscillometric BP monitors midway through the long-term follow-up period in early 2012, so it is unknown whether similar results would be seen in settings where BP is measured using manual sphygmomanometers and auscultation.

### Limitations

This study has some limitations. About 28% of the original study cohort did not attend the 54-month follow-up visit, with similar proportions missing in the TI group and the UC group. However, the similar results in the sensitivity analysis adjusting for variables related to missingness, the similarity in follow-up rates at all time points by treatment group, and the specific reasons for lack of follow-up and their similarity by treatment group did not lead us to doubt the results or the assumption of BPs as missing at random. Also, the EHR data analysis included some BP data on almost all of the original 450 study patients.

## Conclusions

According to this study, intensive interventions like that described herein achieve substantial BP reduction and have sustained effects for 24 months (12 months after the intervention ended). Such BP reductions of this magnitude and duration have the potential to result in clinically important effects on cardiovascular events, even if BP was not different at 54 months. Nevertheless, long-term maintenance of BP control is likely to require continued monitoring and resumption of the intervention if BP increases. More work is needed to determine the content, intensity, and duration of reinforcement that are needed for maintaining intervention benefits over a longer period. The EHR is a promising tool for measuring intervention effects and for detecting deterioration of BP control after initial successful control.

## Notes

Supplement. **Trial Protocol**
