# Supplementary material for: Long-term Outcomes of the Effects of Home Blood Pressure Telemonitoring and Pharmacist Management on Blood Pressure Among Adults With Uncontrolled Hypertension: Follow-up of a Cluster Randomized Clinical Trial
Source: JAMA Netw Open. 2018 Sep 7;1(5):e181617. doi: 10.1001/jamanetworkopen.2018.1617 (PMC6324502; doi:10.1001/jamanetworkopen.2018.1617)
Supplement: Supplement. — Trial Protocol [file jamanetwopen-1-e181617-s001.pdf]

2 R01 HL 090965 (Hyperlink) - Extended Follow-up Study

Manual of Procedures

Table of Contents

Introduction 3

Project Summary 3

Study Procedures 4

Study Timeline 4

Eligibility 4

Recruitment 5

Identifying excluded participants 5

Study Visits 5

Levels of follow-up 6

Recruitment letter 6

Follow-up phone calls 7

Follow-up Recruitment Letter 7

Database management 7

Unresponsive patients 7

Reports generated by RedCap database 8

Scheduling study visits 8

Study Visit Schedule 9

Visit 1 (54 months): For Riverside, Home Clinic, or Home visits 9

Visit 1 for visits conducted at Primary Care Clinic 12

Visit 1 conducted via mail/phone 12

Visit 1 conducted by medical records only 15

Visit 2 (54 months+2 weeks) 15

Cardiovascular Event, Death Records 16

Focus Group Participation 16

Participant Retention .... **Error! Bookmark not defined.**

Data Management and Security 20

Data Quality Plan 20

Data Safety and Monitoring Board 20

Data Security 20
Unanticipated Problems or Adverse Events 21
Appendix A: Recruitment Letter 22
Appendix B: Recruitment Phone Script 24
Appendix C: Follow-up Recruitment Letter 27
Appendix D: 54 Month Questionnaire 28
Appendix E: Focus Group Recruitment letter 36
Appendix F: Focus group facilitation guide 37
Appendix G: Pharmacist Interview guide 39
Anti-hypertensive Medications 42

Introduction

This project is for extended follow-up of participants in the Hyperlink study.

The study will observe participants through 54 months (4.5 years) from baseline to examine the long-term effects of the Hyperlink intervention on blood pressure and cardiovascular events. We will also investigate cost effectiveness and dissemination of the intervention into regular health care practice.

Project Summary

Hypertension affects nearly 70 million Americans, and is effectively controlled in less than half. Because uncontrolled hypertension is a leading cause of stroke, congestive heart failure, renal disease, and myocardial infarction, development of cost-effective and scalable hypertension control strategies has been identified as an urgent national priority. The NHLBI-funded randomized HyperLink trial (Home Blood Pressure Telemonitoring and Case Management to Control Hypertension) has developed and implemented a very effective yet simple two-step non-office-based blood pressure (BP) control strategy: (a) patients measured BP at home using telemonitors that stored and electronically transmitted BP data to a pharmacist via a telephone modem, (b) In periodic telephone visits, pharmacists advised patients on medication adherence and lifestyle, and adjusted antihypertensive therapy under a collaborative practice agreement with primary care physicians. This simple strategy has now been rigorously evaluated in a randomized trial in which a high proportion of patients achieved and maintained BP control at 6, 12, and 18 months following enrollment (71%-72%) compared to usual care (45%-57%, P<.01 at all time points). It achieved systolic BP 11 mm Hg lower at 6 months (P<0.0001), 10 mm Hg lower at 12 months (P<0.0001), and 7 mm Hg lower at 18 months (P=0.004) in the intervention group compared to the usual care group.

The purpose of this proposal is to extend follow-up of the 450 patients enrolled in the HyperLink intervention and usual care groups to achieve three specific aims: (a) provide new data on the long-term durability (b) assess the cost-effectiveness of the Hyperlink intervention, and (c) use mixed methods to identify critical factors for delivering the intervention successfully and translating it into practice. All patients will be invited to attend two research clinic visits to measure BP 5 years following enrollment. Extended data will include assessments of BMI, antihypertensive treatment and adherence, use of home BP monitoring, satisfaction with medical care, and surveillance for clinical cardiovascular events. Data collected at visits will be supplemented with interim BP measures extracted from electronic medical records. Long-term health impact and cost-effectiveness will be quantified using directly observed measures and state-of-the-art microsimulation prediction modeling of cardiovascular events and treatment costs. Focus groups and semi-structured interviews with study subjects, pharmacists, and health system stakeholders will provide additional perspectives on 1) optimizing successful delivery of the intervention, and 2) on strategies and barriers for translation into clinical practice. These results will fill critically important knowledge gaps and provide practical information about the costs and benefits of implementing a this scalable and effective new care

model that holds great promise for improving outcomes for millions of Americans with uncontrolled hypertension.

Study Procedures

Study Timeline

This timeline is a revised version of our originally proposed study timeline. A more task-specific timeline is kept in the project folder in the “Project Management/Project timeline” subfolder.

**Tasks**	Year 1 (Qtr) 2013				Year 2 (Qtr) 2014				Year 3 (Qtr) 2015			
Month	3	6	9	12	15	18	21	24	27	30	33	36
Phase 1 (Study Start-up)												
Finalize extension study MOP												
Obtain IRB approval												
Contact and consent study participants												
Phase 2 (Data collection)												
Patient follow-up visits												
Pharmacist key informant interviews												
Patient focus groups												
Clinical stakeholder interviews												
Phase 3 (Analysis & Dissemination)												
Periodic literature review												
Update analytic databases												
Extract and analyze data												
Preparation of journal manuscripts												
Present results at meetings and submit manuscripts												
Additional dissemination activities												

Eligibility

Inclusion:

- Previously enrolled in Hyperlink (n=450)
- Willing to participate in follow-up visit

Exclusion:

- Deceased
- Previously requested to not be contacted by the study
- Incident dementia, mental illness, or any other condition that would limit ability to give informed consent (new since original Hyperlink 18-month visit

Recruitment

Identifying excluded participants

Prior to beginning recruitment, the study coordinators, project manager, and programmer will review records to identify participants who shall be excluded from the study for reasons of death, previously requesting to not be contacted by the study, and new dementia or mental illness.

> <u>Death:</u> The programmer will review all medical record and claims data for evidence of death among the 450 participants. Patients who are deceased will be marked as excluded in the database for reason of death. Death records will be collected from the state and from medical records where possible (see below section on death records).
>
> <u>Previous study drop-outs:</u> Participants will remain eligible and be contacted even if they were "lost to follow-up" (i.e., missed several visits or stopped responding to our attempts to reach them). Participants who dropped out for other reasons or specifically told us they do not want to be contacted by our study will be excluded from recruitment. The project manager and study coordinators will identify those participants using our old drop-out notes from the original Hyperlink study. The programmer will include drop-out information in the new study RedCap database, and the project manager and study coordinators will use those notes to make decisions about who to the initial recruitment letter to.
>
> Former participants who dropped out for any reason are excluded from Hyperlink2 recruitment.
>
> <u>Incident dementia or mental illness:</u> The programmer will review all medical record and claims data for evidence of new dementia diagnosis or treatment and serious mental illness among the 450 participants. We will make note of participants with new dementia diagnosis so that during the consent process we can assess ability to consent.

<u>Excluding participants:</u> Indicate participant exclusion in RedCap by choosing "excluded from participation" on the Recruitment Form, selecting a reason for the exclusion, and leaving a note about the circumstances. The participant will thus be excluded from further attempts to contact or schedule visits.

Study Visits

There are two study visits:

- Visit 1: Due at 54 months from baseline, +/- 4 weeks.
- Visit 2: Due 2 weeks after Visit 1, +/- 5 days.

Participants should be scheduled within the time window if possible. It is preferred to schedule participants as closely to their 54 month date as possible.

If participants cannot be reached for some period of time, or for any other reason they can only be scheduled outside their time window, we will still schedule their visit and collect their data **<u>up to 6 months on either side of their visit due date.</u>** Participants who cannot attend a visit during that time period will not be included.

Focus group/interview participation will be determined in 2014. See that section of MOP for details.

Levels of follow-up

Participants will be invited to attend the two specified clinic visits at the Riverside Research Clinic. However, because some participants in the original study expressed distress at traveling to Riverside (and thus did not attend study visits), we will offer other options for participating (see Table 2).

These secondary options should ONLY be given if the participant otherwise declines participating at Riverside Clinic. They are also outlined in the Recruitment Phone Script (see next section on Follow-up phone calls and Appendix B).

Important note: If a patient says they do not want to participate, please go through all the levels of follow-up with them prior to considering them "excluded." We would like to ensure patients have the chance to accept the medical records option for follow-up, so we can at least collect EMR data on them later.

Table 2: Levels of follow-up, in order of priority

Location	Visit 1	Visit 2	Measures	Compensation
Riverside (preferred)	X	X	All	$40 V1, $20 V2
Participant's home or primary clinic	X		All	$20 (V1 only)
Mail or phone	X		Questionnaire and medical history only	$10 (V1 only)
Medical Records Only	X		Only medication inventory from EMR	None

Recruitment letter

After excluded participants are identified as excluded in the database, the recruitment letter will be mailed to all remaining participants.

<u>RedCap Invitation Letter report</u>: The report from RedCap will indicate all 450 participants, except for those excluded from participation. The report will list the participants' name, DOB, MRN, and main address. It will also list the participants' previous study dates and the participants' upcoming study visit dates (54 months, 54 months+2 weeks).

Export the report to an Excel file and save in the project folder under "recruitment." The excel file will be used to mail merge the participants' name and address and study dates into the recruitment letter.

The recruitment letter is found under **Appendix A**.

Follow-up phone calls

Timeline: Participants will be called for scheduling when they are within 3 months of their V1 due date. The Upcoming V1 RedCap report indicates upcoming due visits.

Phone script: Follow the Recruitment Phone Script (see **Appendix B**) to discuss the study with potential participants.

Attempts to reach: Keep track of all phone calls placed to and received from patients in the Communication Log. Guidelines on number of calls to place are:

> Calls per week: Unlimited, but use judgment and no more than one call per day
> Voicemails per week: Two voicemails per week, up to two more in following week
> Calls before stopping calling: Up to your judgment, but should stop if have been calling for longer than 2-3 weeks without response (and move onto mailing)

Follow-up Recruitment Letter

Once patient is determined difficult to reach or not answering phone, you can send a second follow-up letter stating that we have been trying to reach the patient for a study opportunity. Please log the second letter into the Recruitment form communication log.

The follow-up recruitment letter is found under **Appendix C.**

Database management

All recruitment letters and phone calls and their outcomes will be logged in the RedCap database under each participant's Recruitment Form.

Unresponsive patients

When the coordinator cannot reach a participant using the initial means of recruitment letter and follow-up phone calls, we must make other attempts to reach the patient. These options should be exhausted before declaring a participant "lost."

> Electronic Medical Record: The coordinator will look in the participant's electronic medical record for any alternative contact information, including phone numbers or addresses, and/or any indication of backup or family contact information. If any alternative information is found, the coordinator will record with notes in the participant's RedCap recruitment form. The coordinator can then re-send the recruitment letter or begin calling the participant as appropriate. In the event of using backup or family contacts, the coordinator should NOT identify the research study as the reason for trying to get ahold of the patient. The coordinator can identify they are calling from HealthPartners, but nothing more specific.

Follow-up letter: After the above means are exhausted, we will send the recruitment letter a second time incase it was originally lost. We will include a cover letter explaining we have made several attempts to reach the participant but have not been able to get ahold of them. (Send both the original recruitment letter and follow-up letter together for this mailing – **Appendices A** and **C**).

Checking death records: Every 6 months, the HPIER programmer will look in the EMR among participants for indication of death. However, upon continued inability to reach participants the coordinator may also look for indication of death in the EMR and through various other means (i.e., searching online databases). See death records section of MOP for record keeping instructions.

Reports generated by RedCap database

The RedCap project offers customizable reports to help us manage patient schedules and progress through the study. We can add reports as desired. Currently, the reports available are:

Introduction letter: To send first letter to all patients. Data included: participant name, study ID, MRN and DOB, participant address and phone number, previous visit dates, and V1/V2 due dates. All visit dates and identifying information will be used for mail merging into the recruitment letter form.

Upcoming V1: Patients due for V1 in 3 months that require scheduling. Data needed: pt name, pt id, address, previous visit dates, and v1/v2 due dates. Patients will be excluded from this list once scheduled for V1.

Upcoming V1 or V2 not scheduled, reached max attempts to call: Needs action as “unresponsive participant.” Patients on this list are those with “not scheduled, reached max attempts - send letter” as call log outcome. Data needed: pt name, pt id, address, v1 or v2 due date (whichever relevant).

Sent letter, bad address: Need to investigate to find new address and re-send letter. Data needed: pt name, pt id

Visit No-Shows or incomplete visits: This list is of participants who scheduled V1 or V2 visits but their V1 or V2 forms remain incomplete. They will require follow-up to re-schedule.

Scheduling study visits

Schedule visits in Epic under appropriate coordinator for following time periods:

- Visit 1 at Riverside: 1 hour
- Visit 1 at home or other clinic: 1 hour +/- 1 hour for travel and setup.
- Visit 2 (always at Riverside): 30 minutes

Visit scheduled dates should be recorded in the Recruitment Form under "Visit 1 date scheduled" or "Visit 2 date scheduled." Visit 2 may be scheduled at time of scheduling V1 or during V1 itself.

Instruct patients to bring all current medications with them to their visit.

Study Visit Schedule

The table below shows the numbers of patients due for follow-up visits at various time points per calendar month, not including those excluded for various reasons.

**2013**				**2014**												**2015**									
**9**	**10**	**11**	**12**	**1**	**2**	**3**	**4**	**5**	**6**	**7**	**8**	**9**	**10**	**11**	**12**	**1**	**2**	**3**	**4**	**5**	**6**	**7**	**8**	**9**	**10**
6	15	29	28	30	30	19	47	19	14	26	18	17	15	11	10	10	15	6	5	16	6	15	15	23	5

All patients will have a 54 month visit (Visit 1). All V1s are due between September 2013 and October 2015 and will occur between August 2013 and November 2015 including the +/-4 week grace period.

All patients attending V1 at Riverside will have a V2 within two weeks of V1 +/- 1 week. Therefore, all V2s will occur between September 2013 and December 2015.

Visit 1 (54 months): For Riverside, Home Clinic, or Home visits

Visit Preparation

Prior to Visit 1, prepare the following:

- Paper chart from Hyperlink 1
- Blank consent form, HIPAA form, and ROI forms
    - Collect up-to-date ROI on all patients for future possible records collecting
- Print medication list from Epic, identify which are anti-hypertnesion meds
- iPad – make sure it's charged, ready to use and logged into RedCap
- Review RedCap Recruitment form for any possible sign of consenting issues (i.e., dementia diagnosis)

Visit Procedures

1. **Informed consent procedure.**
    a. Prior to leaving the patient alone with the consent forms, spend some time talking to the patient about neutral topics and get a sense of their understanding of why they are here today.
    b. For patients with any new dementia diagnosis or other reason to doubt ability to consent, consult one of the clinicians on staff that day. If patient cannot tell you about the study or what they are agreeing to, do not proceed with study visit.
    c. Offer to provide a copy of the signed consent form to the patient if so desired.

2. **Verify/update patient contact information** on paper chart

3. **Medication inventory.**
    a. Ask patient to show you med bottles they brought into the visit.
    b. Ask the patient about each medication listed in their Epic chart, and verify the drug, dose and frequency for each medication.
    c. Make edits on Epic print-out or data collection form.
    d. Verify by looking at bottles, and reconcile any discrepancies between bottles and Epic list so you are sure you have collected accurate drug information.

4. **Cardiovascular history.**
    a. Ask patient questions in the cardiovascular event history section of the data collection form.
    b. For any event to which the patient answers "yes," write down the closest date information, hospital/treating physician and a basic narrative of circumstances on the paper form. This will help us find the records later.
    c. Record information about any event the patient reports, even if it falls outside the window of dates you specifically asked about. We may uncover some previously un-reported events, and want to make sure we have the best self report data possible. When you enter these events into RedCap, you will see previously reported events entered and you will know whether we have that self-report already or not (for events occurring during Hyperlink 1 that may be reported to us here).
    d. When in doubt, record the information and allow the adjudication process to determine whether the event is relevant or not.

5. **Questionnaire.**
    a. Bring up the patient questionnaire on the iPad. Be sure to log out of RedCap and close the original RedCap tab prior to handing the patient their unique web-survey questionnaire. Instruct the patient on how to scroll through the page and tab between pages.
    b. If the patient has problems with the iPad or does not prefer to use it, you may print off a paper questionnaire from the project folder (found under Project Management/Forms and Questionnaires/Approved Materials). Fill out all the pages with the patient ID and date prior to administering.
    c. If the patient cannot complete the survey during the visit for some reason (i.e., needs glasses to read), you may either print off a copy of the patient questionnaire or have the web survey link emailed to them to fill out at home. If you take this option, provide the patient with a stamped envelope to return the paper survey (if paper is chosen). Be sure to indicate this option in RedCap so we can track the surveys sent home and follow up with patient if they do not return it within 2 weeks.

6. **Physical Measures.**
    a. First bring patient out to be weighed and record in lbs on data collection form
    b. Then blood pressure:
        i. Both feet uncrossed and flat on the ground
        ii. Position patient's arm with elbow at heart level. Adjust height of arm using room pillows if needed.
        iii. Wrap cuff over uncovered upper arm with bladder over brachial artery
        iv. Wrap snugly, but not so tight that cuff is restrictive
        v. Explain the body position, quietness and time involved with measurement to patient
        vi. Leave patient alone in room for 5 minutes in proper position
        vii. Return to room to take first reading, wait one minute in between subsequent readings.
        viii. Record all 3 blood pressure readings and the average of three
        ix. Total time for blood pressure protocol is about 12 minutes

7. **Participant compensation and follow-up**
    a. Give patient $40 Target gift card for their time. Record card # on paper chart.
    b. Tell patient about future focus group opportunity and ask whether they think they'd be interested
    c. Schedule Visit 2 for two weeks from Visit 1 date, +/- one week.

Source documents

All data should be recorded onto the data collection form and kept in the patient's study chart, except for the questionnaire which is administered directly on the web survey. Any paper questionnaires administered should be kept in the paper chart, as well.

Electronic data entry into RedCap

- Recruitment Form: Update patient's contact information if needed, enter Visit 2 scheduled date.
- V1 Data Collection Form: Enter all responses to fields included on this form, collected during visit. Matches the paper form including the same questions.
- 54 Month Questionnaire: Should be automatically filled out by patient completing web survey. If paper questionnaire completed, enter responses here.

When a patient's information is complete for each form, change the status of the form from Incomplete/Unverified to Complete.

Epic documentation

The study visit should be documented in Epic as a provider encounter using the Hyperlink visit summary template and accompanying smartsets. The study visit should be copied to the primary care providers' inbox. Contact Deana Grabow for any problems with the Epic documentation.

Visit 1 for visits conducted at Primary Care Clinic

Visit Preparation

If a patient would like to elect this option for follow-up, gather the patient's schedule preference and then consult with Anna so she can contact the clinic CDS to clear the visit. Anna, the CDS, and the scheduling coordinator can work together to schedule the visit and arrange logistics. You'll need a private exam room for about an hour and access to a scale.

Prior to Visit 1, prepare the following:

- Everything the same as visits at Riverside
- Bring a $20 gift card with you for

Visit Procedures

Follow all the same visit procedures as though it were a Riverside visit.

1. **Participant compensation and follow-up**
    a. Give patient $20 Target gift card for their time. Record card # on paper chart.
    b. Patient will <u>not</u> be invited to FG or to a V2 blood pressure check

Source documents and electronic data entry into RedCap

Follow all the same documentation procedures as though it were a Riverside visit.

Epic documentation

You can choose whether to enter the visit/encounter info from the primary clinic or from Riverside. It can be documented as a Riverside Visit. Make note that the visit actually occurred off-site.

Visit 1 conducted via mail/phone

Patients who do not want to come to Riverside or a home/clinic visit can elect to conduct their visit via mail or phone. Phone visits will be less robust than mail visits. At each visit, a smaller subset of data are collected:

Phone Visit Preparation

Phone visits will likely occur at the time you call someone to schedule their V1. Therefore you will not have much prepared, and that's okay. It might be easiest to schedule a phone visit so you have time to prepare, but this can be done on the fly if needed.

Prior to asking any study related questions, you must pause and gather the following:

- Elements of consent document
- Paper forms for the patient questionnaire and the 54 month data collection form
- Patient's epic chart
- Review Redcap Recruitment form for any possible sign of consenting issue (i.e., new dementia diagnosis)

Phone Visit Procedures

1. **Informed consent procedure.**
    a. Prior to proceeding with any questions, review the Elements of Consent document with the patient. If they do not want to hear the whole document read to them, then tell them you will send it to them in the mail so they can review. If they agree to proceed with the study questions, they imply their consent to participate in the study, which they can always withdrawal at a later date.

2. **Verify/update patient contact information** on paper chart.

3. **Medication inventory.**
    a. Ask the patient about each medication listed in their Epic chart, and verify the drug, dose and frequency for each medication.
    b. Make edits on Epic print-out or data collection form.

4. **Cardiovascular history.**
    a. Ask patient questions in the cardiovascular event history section of the data collection form.
    b. For any event to which the patient answers “yes,” write down the closest date information, hospital/treating physician and a basic narrative of circumstances on the paper form. This will help us find the records later.
    c. Record information about any event the patient reports, even if it falls outside the window of dates you specifically asked about. We may uncover some previously un-reported events, and want to make sure we have the best self report data possible. When you enter these events into RedCap, you will see previously reported events entered and you will know whether we have that self-report already or not (for events occurring during Hyperlink 1 that may be reported to us here).
    d. When in doubt, record the information and allow the adjudication process to determine whether the event is relevant or not.

5. **Questionnaire.**
    a. Bring up the patient questionnaire on the iPad. Be sure to log out of RedCap and close the original RedCap tab prior to handing the patient their unique web-survey questionnaire. Instruct the patient on how to scroll through the page and tab between pages.
    b. If the patient has problems with the iPad or does not prefer to use it, you may print off a paper questionnaire from the project folder (found under Project Management/Forms and Questionnaires/Approved Materials). Fill out all the pages with the patient ID and date prior to administering.
    c. If the patient cannot complete the survey during the visit for some reason (i.e., needs glasses to read), you may either print off a copy of the patient

questionnaire or have the web survey link emailed to them to fill out at home. If you take this option, provide the patient with a stamped envelope to return the paper survey (if paper is chosen). Be sure to indicate this option in RedCap so we can track the surveys sent home and follow up with patient if they do not return it within 2 weeks.

6. **Physical Measures.**
    a. First bring patient out to be weighed and record in lbs on data collection form
    b. Then blood pressure:
        i. Both feet uncrossed and flat on the ground
        ii. Position patient's arm with elbow at heart level. Adjust height of arm using room pillows if needed.
        iii. Wrap cuff over uncovered upper arm with bladder over brachial artery
        iv. Wrap snugly, but not so tight that cuff is restrictive
        v. Explain the body position, quietness and time involved with measurement to patient
        vi. Leave patient alone in room for 5 minutes in proper position
        vii. Return to room to take first reading, wait one minute in between subsequent readings.
        viii. Record all 3 blood pressure readings and the average of three
        ix. Total time for blood pressure protocol is about 12 minutes

7. **Participant compensation and follow-up**
    a. Give patient $40 Target gift card for their time. Record card # on paper chart.
    b. Tell patient about future focus group opportunity and ask whether they think they'd be interested
    c. Schedule Visit 2 for two weeks from Visit 1 date, +/- one week.

Source documents

All data should be recorded onto the data collection form and kept in the patient's study chart, except for the questionnaire which is administered directly on the web survey. Any paper questionnaires administered should be kept in the paper chart, as well.

Electronic data entry into RedCap

- Recruitment Form: Update patient's contact information if needed, enter Visit 2 scheduled date.
- V1 Data Collection Form: Enter all responses to fields included on this form, collected during visit. Matches the paper form including the same questions.
- 54 Month Questionnaire: Should be automatically filled out by patient completing web survey. If paper questionnaire completed, enter responses here.

When a patient's information is complete for each form, change the status of the form from Incomplete/Unverified to Complete.

Epic documentation

The study visit should be documented in Epic as a provider encounter using the Hyperlink visit summary template and accompanying smartsets. The study visit should be copied to the primary care providers' inbox. Contact Deana Grabow for any problems with the Epic documentation.

Visit 1 conducted by medical records only

Patients who decline a clinic, home, or phone visit may provide consent for medical record review instead. If a patient consents to medical record review, their Epic record will be reviewed for the following data points:

1. Medication inventory
    a. Use current medication list in Epic
2. Cardiovascular history
    a. Search chart review for any of the relevant cardiovascular events of interest within the specified time range for that patient
    b. Request any supporting records needed for adjudication

Keep all source documentation as described above for other visit types. Record data in the patient's Redcap record for V1 as a 'medical records only' visit type. Complete the form when data entry is done.

Visit 2 (54 months+2 weeks)

The purpose of the 2-week follow up visit (V2) is to take a second set of blood pressure measurements to improve accuracy of the long-term blood pressure outcome. Only patients who attended their 54 month V1 at Riverside Clinic are eligible for V2. Patients electing for their V1 at their home clinic, home, over the phone, or via medical records will not have this blood pressure measurement performed.

1. Physical Measures
    a. Take the patient's blood pressure three times, 1 minute apart, as described in the methods above for V1.

2. Data collection
    a. Confirm whether the patient has changed any medications since their V1. If they have, record all med name and dose changes.
    b. The blood pressure data will be recorded in a separate RedCap form (called V2) for each patient.

There is no questionnaire collected at V2

3. Data entry into RedCap

a. Record any med changes in the V2 form
b. Record each blood pressure and heart rate value
c. Record gift card number dispensed to patient and confirm you have provided a "completion certificate" to the patient
d. Complete the RedCap form once all entry is done

Cardiovascular Event, Death Records

We will collect medical records of all reported events that are collected from the V1 questions. Most of the questions have very clear yes or no answers, but occasionally there will be "gray area" events that are unclear as to whether an event of interest actually occurred.

Please print or request records for all events of interest and re-route to Anna's office at Ceridian. For hospital stays, we need discharge summaries if possible. For ED visits we need an ED summary. For anything involving an office visit, we'd like to see the office visit where the diagnosis was made or event described. If you cannot locate these, please send any documentation you can find that references the incident and we will track down any necessary supporting docs.

As for deaths, we will collect death information for all patients that we learn have died during the study period. Please inform Anna when a death has occurred. If the death occurred during an attempt for recruitment, please mark "excluded" in the recruitment database with the reason being deceased. Please also note all details that you know of surrounding the death so that we can track down the necessary documentation. For patients who you learn have deceased at some time later, please let Anna know. We will be recording all deaths. For death adjudication,

All events and death reports will be reviewed by Karen or Joann to determine the final event category. Anna will coordinate this and keep a spreadsheet of all reports and final adjudications.

Focus Group Participation

Identification and Recruitment of FG participants

We will hold a series of patient focus groups to better understand the reasons some patients were more successful in lowering their blood pressure through this intervention than others. Focus groups will be stratified by: treatment group, blood pressure patterns over time.

We will select focus group participants among those completing a V1 visit. Among those patients, we will sort by treatment group X blood pressure outcomes at 6, 12, 18, and 54 months. The following strata are identified:

Telemonitoring Intervention	Usual Care
1a. Always in control	1. Always in control

1b. Always in control, not long term (relapsed 54m)	
2. Never in control	2. Never in control
3. Achieved control and maintained, late start	3. Achieve control at 12 months
4. Relapsers (mixed)	4. Relapsers (mixed)

Patients falling into these categories will be invited to join a focus group by mail, followed by a phone call from the project manager. See **Appendix E** for focus group recruitment material.

Groups will be scheduled as they fill with at least 4 participants. Ideally groups will have 5-7 participants each.

Focus Group guide
See **Appendix** F for final focus group facilitation guides.

Conducting the Focus groups

Procedures for conducting the focus groups are as follows:

Purpose
The purpose of these discussion groups is to learn why some participants had better results with Hyperlink than others in lowering their BP. We have selected groups of participants based on how their blood pressure changed over time during the intervention and long after.

Facilitator: Anna
Co-facilitator/assistant: Ann Tucker, Julie Anderson, Sarah Basile, Amy LaFrance

Preparations ahead of group (week before/day before)

- <u>Gift cards</u>
    - $50 Target gift cards will be purchased and kept in Anna's locked desk at Riverside. All the serial #s will be tracked in this spreadsheet: (make spreadsheet). Prior to group, cards will be recorded with date and participant's studyID they are given to in that sheet.
- <u>Scheduling Riverside conference rooms</u>
    - To schedule a conference room, contact:
    - Book the room an hour prior to group starting and 30 minutes after ending, if possible (at least 30 minutes on either side)
    - Ensure there are proper plugs accessible in the chosen conference room
- <u>Food</u>
    - Jimmy Johns online www.jimmyjohns.com
    - Order 15-piece party platter with mix of sandwich types ($26.99) and one cookie tray ($19.99). Delivery fee is $4. Total food cost per group: $54.46
    - Cups available from Riverside Clinic. Designate some pitchers for ice/water.
- <u>Materials</u> – *all will be stored in Anna's desk at Riverside*

    - Participant consent forms are stored in Anna’s desk in a purple folder. Here is the original version for printing if needed: Focus Group Materials\IRB Materials\IRB Approved FG Materials\IRB approval of FG materials and consent form.pdf
    - Nametag stickers
    - Pens for consenting and sharpies for nametags
    - Parking passes

Day of group

- 1 hour prior:
    - Set up signs in lower level. Obtain easel from Sherry Cole in administration and place past registration desks to point patients to the Falk rooms.
    - Set up recording equipment and laptop in conference room
    - Arrange tables appropriately for group
- 30 mins prior: Jimmy John’s delivered. Fill up pitchers with ice and water. Make coffee on lower level.
- As participants arrive:
    - Greet participants, shake hands and introductions
    - Have them fill out a nametag
    - Give them a consent form and tell them to review it while they have some food and get settled in
    - One by one, Anna will call participants over to a private area to talk with them and review and sign the consent form
    - A participant roster will be created for each group listing who is present. Names can be checked off as they arrive.

Group will start promptly at the designated hour

During the group: note taking

- The co-facilitator’s main role during the group will be taking notes. Notes will be attached to the file with the group roster and brought up on Anna’s computer.
- Format of notes:
    - Notes can be formatted however the notetaker prefers. Speakers can be identified by first names or by initials or speaker #s. Please just make a note of how you refer to each participant in the notes so we can de-identify them easily later.
    - Please also periodically record the time in the notes so we can match them up with the audio recording and transcript
    - Notes should be as close to verbatim as you can get, given so much talking. If you can’t get it word for word, please try to get as close to as possible and indicate where you’re not sure what was said. You can paraphrase by leaving words out, but please do not construct meaning or interpret what the participant is saying when you record the notes.

- You can provide your own interpretation, further questions, or comments as an aside in parentheses if you choose to do so.

During the group: facilitation

- Anna will start out the group with a welcome and a request for everyone to respect the privacy of the other participants by keeping what is discussed in this room private to the room.
- We will lead introductions around the room with participants name, where they are from, and their favorite thing about this past summer (or another ice breaker)
- Anna will facilitate the group entirely. If the note taker has more reflections or questions, we will wait until the end of the group to go back to them to allow space for things to arise spontaneously from the participants.

After the group

- Each participant should receive a gift card and parking pass
- Recording will be confirmed
- Note taker and Anna will sit down to de-brief and go over the notes, identify themes that stood out and questions that seem interesting to follow up more on. Should also make any relevant notes on certain participants and where they seem to be coming from, to help with interpretation of transcripts in the future.

Co-facilitator/note taker should effort time spent on this group to Hyperlink2 x1207900

Pharmacist Interviews

We will conduct in-depth interviews with each telemonitoring intervention pharmacist (n=4) for the purpose of understanding factors in patients'' success according to their clinical point of view.

Each pharmacist will be invited for an interview and will receive a thank-you gift card. Two of the intervention pharmacists no longer work for HealthPartners but will be interviewed by phone.

Interview guide

See **Appendix G** for the final pharmacist interview guide.

Conducting the interview

Interviews will be conducted by project manager Anna. The interview guide will be followed and the interview will be audio recorded. Interviews will be conducted in the MTM's office or by phone. After the interview is complete, audio recordings will be transcribed and the audio file and transcription saved on the project drive. All interviewees will receive a $50 gift card for participating.

Data Management and Security

Data Quality Plan

Data will be reviewed quarterly starting in Jan 2014. Our primary concern is with:

- Data completeness (missing visits and missing values)
- Visit timeliness (in window/out of window/avg. time of followup)
- Overall patient flow (pt dispositions).
- We can also tabulate frequencies and means on key variables to look for outlying/implausible values.

The flow diagram is constructed of elements we already collect in RedCap in the Study Progress Report. We will update this with each quarter for presentation to the group in Jan, April, July, and October of each year.

Data Safety and Monitoring Board

There is no DSMB for this project, as the study is only observational and does not have an intervention component.

Data Security

iPad security

iPads will be stored in locked cabinets in the Riverside Clinic. Only coordinators on the Hyperlink project will have access to the iPads. They will be secured by HealthPartners IS&T and in the event they are misplaced, stolen, or otherwise not in our possession will be wiped clean of all data. There should not be any PHI stored on the iPads. Only RedCap should be accessed via the internet and the coordinator's password to the RedCap website should be entered each time, not saved for automatic log-in.

At the conclusion of the study, iPads will remain in possession of the Riverside Clinic until requested to be returned by NHLBI.

RedCap access

Access to the Hyperlink RedCap project is restricted to: Riverside coordinators collecting Hyperlink data, project manager, statististican, and programmer who created the RedCap project record.

Qualitative data recordings

All audio recordings and transcriptions will be kept in a project drive folder protected by password and drive access permission.

Unanticipated Problems or Adverse Events

Because this is an observational study without intervention, we are not systematically monitoring patients for medical events potentially related to hypertension treatment. We will, however, record all self-reported cardiovascular events as described above which will be assessed for potential relationship to hypertension treatment. Self-reported data will be supplemented with claims and medical records at the conclusion of the study.

Any unanticipated problems related to patient safety, privacy, or other concerns will be reported to the IRB if applicable.

Appendix A: Recruitment Letter

Dear [Participant's Name],

I am writing to thank you for your participation in Hyperlink, the blood pressure study you were enrolled in for about 18 months between 2009-2012.

You might recall that the purpose of the Hyperlink study was to test a program for blood pressure management that involved using a home blood pressure telemonitor and receiving support from a pharmacist over 12 months. About half the participants received that program, and the other half worked with their doctors like they normally would. Everyone in the study was invited to an enrollment visit at the HealthPartners Riverside Research Clinic and then to follow-up visits at 6, 12, and 18 months after enrollment. We are happy to report that we have now completed all those visits, and we are thankful for your participation.

The Hyperlink study was quite successful in gathering new information about blood pressure management. Our short-term results suggest the home monitoring and pharmacist support combination was effective in helping people lower their blood pressure. We will be sharing the official results with you soon via newsletter, so please watch for that information.

It is important for us to know if the short-term results translate to long-term effects, so we have applied for and received funding to conduct a follow-up study to continue to observe your blood pressures through up to 54 months (4.5 years) from enrollment. **Therefore, we would like to invite you to continue your participation in Hyperlink by attending two further visits at our Riverside Research Clinic.**

Everyone is invited to come back for one visit at 54 months (4.5 years) after enrollment, and a second visit two weeks later. Below are the dates of your previous Hyperlink visits, and your potential future visit dates:

Enrollment	
6 months	
12 months	
18 months	
**54 months**	
**54 months + 2 weeks**	**2 weeks later (BP check)**

The first visit would involve taking your blood pressure and filling out questionnaires similar to what you did in the past. The second visit would be only a simple blood pressure check. There will be no labs with these visits, and you will receive a $40 gift card for the 54 month visit and a $20 gift card for the second visit (BP check). Participation in this study does not involve any additional home monitoring or pharmacist services – you will just continue taking care of your blood pressure like you normally would. Finally, you may also be invited to provide feedback about your experience in Hyperlink through focus groups or interviews. You will be notified separately if selected for that part of the study. Because these visits were not part of our original plan, we will have a new consent form for you to review and sign that will explain other details of the new study elements.

You will receive a phone call from our Riverside Research Clinic staff about three months prior to your 54 month visit to discuss the opportunity further and answer any questions. If you'd like, you can also call us at 612-341-1950. If you do not wish to participate in the long-term study, you can notify us by calling that number.

Thank you for thinking about the study. Your participation in Hyperlink has been so valuable, and we look forward to continuing to work with you in the future.

Warm wishes,

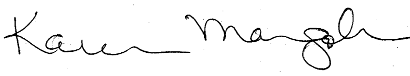

Karen Margolis, MD, MPH
Hyperlink Principal Investigator
HealthPartners Research Foundation

Appendix B: Recruitment Phone Script

Hyperlink Extended Follow-up
Recruitment Phone Script

Note: This script will appear for each patient in a "recruitment" section of our database, likely in RedCap. Fields will be entered using either text or check-boxes.

Patient Name:
Gender:
DOB:
MRN:
Phone #1:
Phone #2:

Call attempt #: (*1, 2, 3, 4, 5*)
Date/time of call:
Outcome of call: (*No answer, left message, successful contact*)

Researcher: Hello, may I please speak with (Participant's name)?
Non-participant answering: No, he/she is **not** home.
Researcher: What would be a good time for us to callback? *(Record notes in recruitment database, with suggested date/time for return call)*

Participant: **Yes**, this is (Participant).
Researcher: Hi (Participant), this is (Research coordinator) and I am calling from HealthPartners Riverside Research Clinic with the Hyperlink study. How are you today? *(Wait for answer)*

In August 2013, we mailed you a letter about a follow-up study we are conducting to Hyperlink. Did you receive that letter? *(Wait for answer, proceed to explain what was in the letter whether they got it or not)*

We are interested in inviting you back to another two visits to check your blood pressure and ask you some more questions. For you, those visits would be due around [**54 month visit date**], and then two weeks after that. We are interested in finding out if the results from Hyperlink last for a long time or not. You would get paid $40 for each of those visits. We also will select certain people to invite to be in focus groups to tell us about their experience in Hyperlink so we can figure out how to make it work for everyone. That would be later on in the study, though and would be optional for those invited.

Does this opportunity interest you at all?

Participant: →**No**.

Researcher: May I ask why? We want to make it easy for everyone to join.

Participant: *(Inconvenient, too far away, don't like driving to Minneapolis, etc)*

Respondent: Would it work better for you if we could meet you at your regular clinic to conduct the visit? You would get a free blood pressure check that way, and you could still participate without the hassle.

Participant: → **Yes**, that would work.

Researcher: Great. You would be due for the first visit on (*date*). Can we call you closer to that date to schedule? (*If yes, say thank you and record info. If it's already close to the date, move on to scheduling the first visit*).

Participant: → **No**, I don't want to come in for any visits.

Researcher: Okay, I understand. Would you be interested in answering any questionnaires by mail? They would be the same you'd fill out at the visit, except we'd mail them to you with an envelope to return them by mail. It would be one packet per year, and would take about 30 minutes to fill out total. Alternatively we could just call you and ask you the survey questions over the phone when your due dates come. That would also take about 30 minutes and you'd still receive the $40 gift card for each survey you answer.

- ➔ **P: Sure**, the MAIL option sounds fine.
  - R: Great! I will note that here, and you will receive a packet in the mail around your due date. If we don't hear from you after about 3 or 4 weeks we'll call you to make sure you received it. *(Confirm best phone number to use, mailing address, etc. Make notes in recruitment database)*
- ➔ **P: Sure**, the PHONE option sounds fine.
  - R: Great! I will note that here. Do you have a certain time of day that is best to call you? Would you prefer us to schedule a specific 30 minute period with you to conduct the phone surveys? *(Confirm best phone number to use, time to call, mailing address for gift cards, etc. Put on schedule if necessary. Make notes in recruitment database)*
- ➔ **P: No**, I don't want to do either of those.
  - R: Fair enough. We certainly understand. Even if you prefer not to make visits or communicate with us by phone/mail, it would still be very helpful to the study if we were able to check your HealthPartners medical records. We would look for blood pressures from your doctor visits, blood pressure medicines, and other cardiovascular problems if you have any. The information would be kept strictly confidential and not shared with anyone outside

the study, and your name will be separated from the information. Would you be able to give us permission to look at your medical records during the study time period in order to look for hypertension related information?

- ➔ **<u>P: Yes</u>** that's fine.
  R: Thank you very much, and thank you for your time. Have a great day!
- ➔ **<u>P: No</u>**
  R: Okay. We will respect your wishes and not use your information for this follow-up period. If you ever change your mind, feel free to give us a call back and let us know. Thanks again for being in Hyperlink!

Participant: *(No interest in being in more research)*
Researcher: *(Offer same options as above in same order: option to participate via mail, option to consent to medical records)*

Outcome of recruitment attempt: (*Not interested, verbally consented to med records only, surveys via mail, surveys via phone, clinic visit at Riverside, clinic visit option for elsewhere*)

Scheduled: (*Schedule in future, Successfully scheduled*)

Best time to call (for phone visits):

Notes: (*open text box for any relevant notes*)

Appendix C: Follow-up Recruitment Letter

Note: This letter should be sent to participants if unable to reach after two attempts at sending the initial mailing to their home and calling for at least two weeks.

Dear [Participant's Name] or family member,

I am writing because my study team has been trying to reach you to discuss your participation in Hyperlink, the blood pressure study you were enrolled in for about 18 months between 2009-2012. We are inviting Hyperlink participants continue in the study by attending one or two more short clinic visits. These visits would help us understand the longer-term benefits of Hyperlink.

We have been unable to reach you either by phone or mail, but would greatly appreciate a response from you.

Please call us at 612-341-1950 and mention the Hyperlink study to discuss this opportunity with one of our research coordinators, Rachel or Ann.

If you would like to decline participation for any reason, you may call and inform us and we will not continue to contact you. If you are unable to respond to us for some reason, you may also have a trusted family member do so on your behalf.

Thank you and be well,

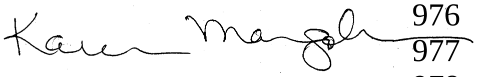

Karen Margolis, MD, MPH
Hyperlink Principal Investigator
HealthPartners Institute for Education and Research

Appendix D: 54 Month Questionnaire

SECTION A- Health Habits

1. During an average week, how many days have you done the following types of activities?

a. Vigorous or very hard physical activity that causes your heart to beat much faster than normal. It is hard to hold a conversation during this kind of activity. **Examples include: running, aerobics, tennis, swimming laps, cross-country skiing, aerobic machine at a fast pace, vigorous bicycling.**

In the past month, how many days each week did you do vigorous physical activity for **20** minutes or more?

0	1	2	3	4	5	6	7
☐	☐	☐	☐	☐	☐	☐	☐

b. Moderate physical activity causes your heart to beat faster than normal. Most people can hold a conversation during this kind of activity. **Examples include: Fast walking, dancing, easy bicycling, easy swimming.**

In the past month, how many days each week did you do moderate physical activity for **30** minutes or more?

0	1	2	3	4	5	6	7
☐	☐	☐	☐	☐	☐	☐	☐

c. Light physical activity causes your heart to beat slightly faster than normal. **Examples include: easy walking, yoga, bowling, golf.**

In the past month, how many days each week did you do light physical activity for **30** minutes or more?

0	1	2	3	4	5	6	7
☐	☐	☐	☐	☐	☐	☐	☐

d. In the past month, how many days each week did you do activities to increase muscle strength, such as lifting weights or calisthenics?

0	1	2	3	4	5	6	7
☐	☐	☐	☐	☐	☐	☐	☐

2. Have you smoked any cigarettes in the past 30 days?

- ☐ Yes
- ☐ No

3. Over the past 30 days, how many cigarettes did you usually smoke each day?

- ☐ Less than 1
- ☐ 1-4
- ☐ 5-14
- ☐ 15-24
- ☐ 25-34
- ☐ 35-44
- ☐ 45+
- ☐ I did not smoke cigarettes

4. Have you consumed any alcoholic beverages in the past 30 days?

- ☐ Yes
- ☐ No

5. Over the past 30 days, how many days per week did you consume alcoholic beverages?

- ☐ 0-1
- ☐ 2-3
- ☐ 4-6
- ☐ 7
- ☐ I did not consume alcohol

6. Over the past 30 days, how many alcoholic drinks did you consume each week?

- ☐ 0-3
- ☐ 4-6
- ☐ 7-12
- ☐ More than 12
- ☐ I did not consume alcohol

7. How frequently do you add salt to your food after it is served at the table?

- ☐ Rarely
- ☐ Several times per week
- ☐ About once a day
- ☐ With almost all meals

8. How frequently do you add salt when preparing or cooking your food?

- ☐ Rarely
- ☐ Several times per week
- ☐ About once a day
- ☐ With almost all meals

9. In the past 6 months, have you made any lifestyle changes as part of your treatment for hypertension? (Mark all that apply):

- ☐ Low salt diet
- ☐ Other dietary changes (such as DASH diet, ate more fruits and vegetables)
- ☐ Cut back on alcohol
- ☐ Weight loss
- ☐ Increased physical activity
- ☐ I did not make any of these lifestyle changes

SECTION B - Your health care

The following questions ask you to rate your confidence in your ability to do certain things that relate to health care and managing your blood pressure. In these questions, health care could be received in person, by phone, or a secure email message. Health care includes taking medications that were prescribed for you.

**How confident are you that you can:**	Not confident<br>1	2	3	4	Very confident<br>5
10. Ask a question of a nurse or pharmacist, even if they are busy?					
11. Talk to a nurse or pharmacist when you have concerns about your treatment?					
12. Get a nurse or pharmacist to fully respond to your questions to your satisfaction?					
13. Contact a nurse or pharmacist from home when you have a question or concern?					
14. Ask questions of your health care team at the clinic, even if they are busy?					
15. Talk to your health care team when you have concerns about your treatment?					
16. Get your health care team to fully respond to your questions to your satisfaction?					
17. Contact your health care team from home when you have a question or concern?					
18. Include checking your blood pressure at home in your weekly routine?					
19. Include your taking medication in your daily routine?					
20. Stick to taking medication even when your daily routine changes?					
21. Afford your medications?					
22. Keep your blood pressure under control?					

SECTION C - Satisfaction with your health care

The following questions ask about your satisfaction with your health care and health care providers. For the following questions, a health provider could be a doctor, nurse, nurse practitioner, clinical pharmacist, or any other person from whom you receive health care. Answer “unable to assess” if you didn’t have enough interaction with health providers to answer the question.

	Never	Some-times	Usually	Always	Unable to assess
23. In the past <u>6 months</u>, how often did your health providers listen carefully to you?	☐	☐	☐	☐	☐
24. In the past <u>6 months</u>, how often did your health providers explain things in a way that you could understand?	☐	☐	☐	☐	☐
25. In the past <u>6 months,</u> how often did your health providers show respect for what you had to say?	☐	☐	☐	☐	☐
26. In the past <u>6 months</u>, how often did your health providers spend enough time with you?	☐	☐	☐	☐	☐
27. In the past <u>6 months</u>, how much of a problem, if any, was it to get the care, tests, or treatment you or a doctor believed necessary?	☐	☐	☐	☐	☐

28. Using numbers 1-5, where 1 is the worst health care possible and 5 is the best health care possible, what number would you use to rate <u>all your health care</u> in the last <u>6 months</u>?

Worst 1	2	3	4	Best 5
☐	☐	☐	☐	☐

29. Using numbers 1-5, where 1 is the worst hypertension care possible and 5 is the best hypertension care possible, what number would you use to rate <u>your hypertension care</u> in the last <u>6 months</u>?

Worst 1	2	3	4	Best 5
☐	☐	☐	☐	☐

SECTION D – Your blood pressure

30. Do you currently take any medication for your hypertension (high blood pressure)?
   - ☐ Yes
   - ☐ No (Skip to Question #31)

   a. Do you ever forget to take your blood pressure medicine?
      - ☐ Yes
      - ☐ No

   b. Are you careless at times about taking your blood pressure medicine?
      - ☐ Yes
      - ☐ No

   c. When you feel better do you sometimes stop taking your blood pressure medicine?
      - ☐ Yes
      - ☐ No

   d. Sometimes if you feel worse when you take your blood pressure medicine, do you stop taking it?
      - ☐ Yes
      - ☐ No

   e. Have you skipped doses of blood pressure medicine in the past month?
      - ☐ Yes
      - ☐ No

31. In the last 6 months, did you ever measure your blood pressure using a home blood pressure monitor?
   - ☐ Yes
   - ☐ No

32. In the last 6 months, on average, how many times did you measure your blood pressure using a blood pressure monitor at home?
   - ☐ Less than once a month
   - ☐ A few times a month
   - ☐ Once a week or more
   - ☐ I did not do this

33. When you used a home blood pressure monitor in the last 6 months, did you share your blood pressure measurements with a health care provider like a doctor, nurse, or pharmacist? (Mark all that apply)
   - ☐ Yes, I wrote my blood pressure numbers down on paper to share

- ☐ Yes, I read my blood pressure numbers verbally to them over the phone
- ☐ Yes, I sent my blood pressure numbers via e-mail
- ☐ Yes, I sent my blood pressure numbers electronically by Smartphone, computer, or telemonitor
- ☐ Yes, I shared my blood pressure numbers with a provider some other way (please specify): ____
- ☐ No, I did not do this

34. In the last 6 months, have you spoken with a pharmacist about managing your hypertension? (Mark all that apply)

- ☐ Yes, when picking up medicine at the pharmacy
- ☐ Yes, I had an appointment with a clinical (MTM) pharmacist by phone or in the clinic
- ☐ Yes, in some other environment (please specify): ___________________________
- ☐ No, I did not do this

SECTION E- About You

35. Which of the following best describes you current paid work status?
- ☐ Working full time
- ☐ Working part time
- ☐ Retired
- ☐ Not currently working

36. Approximately how many hours do you work in a week? (For example, if you work 38 hours on average, you would enter ‘3’ in the first column and ‘8’ in the second column)

☐	0	☐	0
☐	1	☐	1
☐	2	☐	2
☐	3	☐	3
☐	4	☐	4
☐	5	☐	5
☐	6	☐	6
☐	7	☐	7
☐	8	☐	8
☐	9	☐	9
☐	More than 100 hours/week		

37. About how much money do you estimate that you make <u>in an hour</u>? (Pick the choice that is closest).
- ☐ Less than $10/hour
- ☐ $10/hour (would be $20,000/year)
- ☐ $15/hour (would be $30,000/year)
- ☐ $20/hour (would be $40,000/year)
- ☐ $25/hour (would be $50,000/year)
- ☐ $30/hour (would be $60,000/year)
- ☐ $35/hour (would be $70,000/year)
- ☐ $40/hour (would be $80,000/year)
- ☐ $45/hour (would be $90,000/year)
- ☐ $50/hour (would be $100,000/year)
- ☐ Greater than $50/hour

Appendix E: Focus Group Recruitment letter

(Date)

Dear (*participant*),

We are writing to thank you for your participation in the Hyperlink study. You have recently completed a long-term follow-up visit at Riverside Clinic, around 5 years after your original Hyperlink visit. Your dedication to our research is greatly appreciated!

Because you have stayed with our study for so long, we would like to invite you to participate in a discussion group about blood pressure and blood pressure management.

The discussion group would be held in the late afternoon or early evening on a weeknight at Riverside Clinic and would last about 60-90 minutes. The discussion group would be made up of other Hyperlink participants similar to you in some ways.

We are inviting you to this discussion group because we value your insight about blood pressure management and believe we have more to learn from you. The information we learn from these discussion groups will advise further development of our programs to support patients in achieving healthy blood pressure.

The group we are inviting you to would be held on: (*insert date and time options here*)
You will receive a gift card to Target of $50, refreshments, and free parking as a thank you for your participation.

We will be calling you in the next few days to discuss this opportunity further! You can also feel free to call Anna Bergdall, the Hyperlink study Project Manager, at **952-967-5101** to express interest or decline.

Thank you, and we look forward to talking more soon!

Dr. Karen Margolis

Appendix F: Focus group facilitation guide

Focus Group Discussion Guide (Intervention groups)

Prior to group beginning: Each patient should meet with a coordinator to be individually consented, receive a nametag and offered refreshments.

Introduction

- Names/introductions
- Thank you for being here
- Why we are here/purpose of discussion
- Ground rules, encouraging discussion, etc

I'd like to begin by talking about high blood pressure in general. Could you tell me about the kinds of things that affect blood pressure in your experience?

Could you tell me about… (sub-questions if topics do not arise spontaneously)

-What kinds of things do you do now to help your BP?

-What makes it hard to keep your BP in a healthy range?

-How easy or difficult do you find it to take care of your BP?

-History of high BP

<u>Probes</u>: Could you tell me about a time when your BP was healthy/unhealthy? What do you feel helped you get it into a healthy range? Could you tell me about a time when you especially struggled? Why do you think that was?

<u>Dimensions</u>: Medication use and adherence; health care providers; lifestyle efforts; home monitoring; psycho-social stress; social/family environment; self-efficacy

Now let's think back to the time that you were coming to this clinic for the Hyperlink research study. I'd like to hear about your experience with your blood pressure during that time.

-What was most helpful for you then?

-Did you still have challenges with your BP during that time? If so, why do you think so?

-What kinds of things could be changed to make it better for you?

<u>Probes</u>: How did you feel about working with the pharmacist? What about taking your BP at home? What about those things did you like or dislike? Why? (only if pharmacist or home BP were already mentioned). How did those things change other areas of your health? How did those things help you with other efforts, like stress, lifestyle, talking with your health providers, etc? (refer to items mentioned above)

<u>Dimensions</u>: Medication adherence, Rx changes, getting new Rxs; using home BP monitor, adherence, using new technology; relationships with pharmacists; congruence of intervention with other health care; lifestyle efforts; social/family environment; self-efficacy; KEY challenges and KEY factors for success.

Wrap-up: Is there anything anyone would like to add to or clarify, or does anyone have any questions?
Thank you for taking the time to share your experiences with us. We value your input and take your feedback very seriously. We are all looking forward to helping to create better solutions to help people with their blood pressure.

Focus Group Discussion Guide (Usual Care groups)

Prior to group beginning: Each patient should meet with a coordinator to be individually consented, receive a nametag and offered refreshments.

Introduction

- Names/introductions
- Thank you for being here
- Why we are here/purpose of discussion
- Ground rules, encouraging discussion, etc

I'd like to begin by talking about high blood pressure in general. Could you tell me about the kinds of things that affect blood pressure in your experience?

Could you tell me about… (sub-questions if topics do not arise spontaneously)

-What kinds of things do you do now to help your BP?

-What makes it hard to keep your BP in a healthy range?

-How easy or difficult do you find it to take care of your BP?

-History of high BP

Probes: Could you tell me about a time when your BP was healthy/unhealthy? What do you feel helped you get it into a healthy range? Could you tell me about a time when you especially struggled? Why do you think that was?

Dimensions: Medication use and adherence; health care providers; lifestyle efforts; home monitoring; psycho-social stress; social/family environment; self-efficacy

Now let's think back to the time that you were coming to this clinic for the Hyperlink research study. I'd like to hear about your experience with your blood pressure during that time.

-What was most helpful for you then?

-Did you still have challenges with your BP during that time? If so, why do you think so?

-What kinds of things could be changed to make it better for you?

Probes: What kinds of health care providers were you working with at that time? What kinds of efforts were you doing on your own to help your BP? Did your health care providers help you overcome challenges you faced (like stress, lifestyle challenges)? What about working with your providers was difficult?

Dimensions: Medication adherence, Rx changes, getting new Rxs; using home BP monitor, adherence, using new technology; relationships with pharmacists; congruence of intervention with other health care; lifestyle efforts; social/family environment; self-efficacy; KEY challenges and KEY factors for success.

Wrap-up: Is there anything anyone would like to add to or clarify, or does anyone have any questions?
Thank you for taking the time to share your experiences with us. We value your input and take your feedback very seriously. We are all looking forward to helping to create better solutions to help people with their blood pressure.

Appendix G: Pharmacist Interview guide

Introduction

- Introduction/names
- Thank you for being here

Purpose of this interview

The purpose of this interview is to gather your reflections on being an intervention pharmacist for the Hyperlink study. We are in the process of talking to patients about their experience in Hyperlink for the sake of trying to understand what worked well in the study and what didn't work so well to help patients get their blood pressure under control, and why. Your clinical perspective as an intervention pharmacist is helpful also, so we can ultimately understand how to better target this type of intervention for patients in a practice setting.

Let's start by talking about what MTM is. Could you please explain in your own words what an MTM pharmacist is and how it differs from other types of pharmacists?

Probes: What are the credential/degree requirements? What is your prescribing power like? How is an MTM pharmacist differentiated in the clinic setting from the pharmacist one talks to when getting a med filled? How do you think patients understand your role as a pharmacist?

Dimensions: Scope of work; patient perceptions

What is your day to day work like, and what kinds of patients do you work with?

Probes: What is the nature of work you do with your patients; What is the structure of their care within this clinic; How are patients referred to you (self-referral vs. referral by clinicians or disease management); Can you describe your relationship with specialty care or primary care? How do you interact with physicians? Are you currently working with any patients doing home monitoring of chronic conditions, and if so, what?

Dimensions: Relationship to primary care team; regular daily functions of MTM care

Could you describe your recollections of your experience working with Hyperlink patients?

Probes: Could you tell me about what you enjoyed about it? What do you recall being difficult/challenging? Could you tell me about a particular patient that sticks out in your mind and why?

Dimensions: Study protocol vs. practice (specific feedback); relationships with patients; relationships with PCPs

What are your opinions or ideas about why some people were more successful in lowering their BP with this intervention than others?

Probes: What makes some patients difficult for this kind of intervention? What do you feel helped patients the most? What did a very successful patient look like to you? What were some common "sticky points" for patients, presenting barriers? Can you tell me about your thoughts of med intensification vs. telemonitoring? – Specific patient examples come to mind? Typologies? How did your first impression of a patient hold up over time?

Dimensions: Med adherence; lifestyle efforts/counseling; relationship with patients; psycho-social stress; self-efficacy; barriers and facilitators; home monitoring equipment; PCP relationship; clinical judgment

Could you describe in your clinical judgment what you believe the most important "active ingredients" are to this type of intervention?

Probe: Do you see any difference in effectiveness of medication intensification versus telemonitoring? What about patient's time of involvement: what do you think is the optimal time? Do you see any use in continuing to counsel or meet with patients after they achieve control? How long do patients really need with this kind of program to see the best results?

Dimensions: Targeted intervention; medication intensification vs. telemonitoring; active ingredients

You may recall there was a fairly specific protocol in place for this study: Phone visits every two weeks unless the patient was under control, assessments of adherence at each visit, and medication-related actions to be taken at certain visits, depending on the patient's blood pressure and time in the study. Can you tell me about how that protocol worked for you in practice?

Probes: Can you tell me about which pieces of the intervention you made sure to follow protocol on, and which you felt you didn't always follow? What pieces did you adjust to fit more consistently with your regular practice? How did the study protocol prompt different actions than you would have taken without the study? How did you use the AMC website where you went to see the BP readings? What was challenging for you about that protocol? What parts did you feel were most essential for providing the best BP care for your patients?

Dimensions: Study protocol vs. practice; treatment titration protocol; adherence assessments; 3rd party BP data

Can you tell me about the relationship you had with the Primary Care Providers during this study?

Probes: How did you interact with primary care physicians? How does the relationship you had with PCPs during the study look similarly or different to your day to day MTM work? What type of communication do you have with PCPs around specific patients' care? What are some specific barriers to doing so? How do you think this communication affects patients' care? How do you think your work with PCPs can help patients feel like they have a cohesive care team working with them? do you think patients perceived of you versus their primary care? How are most patients referred between MTMs and PCPs (self or outside, vs. pcp referral)? When does this collaboration need to be strong and when doesn't it? This study served as a connecting point between pharmacists and docs/system, how can patients be more connected between all?

<u>Dimensions</u>: Team-based care; communication with PCPs; communication/relationship with patients; referral channels;

What ideas do you have about offering this intervention to patients in our clinics in a non-study setting?

<u>Probes</u>: If you could create your own intervention of this kind for patients with uncontrolled blood pressure, what would it look like? What elements of the Hyperlink intervention are in your opinion essential? What would you change (or remove or add)? Do you have ideas to make this kind of program work for people who had a difficult time in Hyperlink? What do you see as major barriers to implementing this kind of program into day to day pharmacy practice? What about facilitators? What about in a practice setting that is different from HealthPartners with less infrastructure?

<u>Dimensions</u>: Targeted intervention; active ingredients; implementation and uptake

Is there any other feedback you received from patients that you would like to share with us about Hyperlink?

<u>Probes:</u> Can you think of any patients who exemplify the success or difficulties we talked about today? If so, do you think they would be useful to interview (i.e., do you think they could share insights with us we haven't already heard?)

Wrap-up: Is there anything else you'd like to add to or clarify, or do you have any questions?

Thank you for taking the time to share your experiences with us. Your input as an interventionist is highly valuable as we move forward with creating this type of intervention for our patients. If you have any other feedback after we are done we would love to hear from you.

Anti-hypertensive Medications

ACE Inhibitors
Benazepril (Lotensin)
Captopril (Capoten)
Cilazapril (Inhibace)
Enalapril (enalaprilat, Vasotec)
Fosinopril (Monopril)
Lisinopril (Prinivil, Zestril)
Moexipril (Univasc)
Perindopril (Aceon, Coversyl)
Quinapril (Accupril)
Rampiril (Altace)
Trandolapril (Mavik)

Aldolsterone Antagonists
Eplerenone (Inspra)
Spironolactone (Aldactone)

Angiotensin Receptor Blockers (ARBs)
Azilsartan (Edarbi)
Candesartan (Atacand)
Erposartan (Teveten)
Irbesartan (Avapro)
Losartan (Cozaar)
Olmesartan (Benicar)
Telmisartan (Micardis)
Valsartan (Diovan)

Anti-adrenergic Agents (Alpha blockers)
Clonidine (Catapres, Catapres-TTS, Jenloga, Kapvay, Dixarit) – *May have other non-HTN uses (ADHD, Tourette, Opioid withdrawal, menopausal flushing, smoking cessation, alcohol withdrawal)*
Doxazosin (Cardura, Cardura XL) – *May be used for BPH (enlarged prostate)*
Methyldopa (Aldomet)
Prazosin (Minipress)
Terazosin (Hytrin)

Beta-blockers
Acebutolol (Sectral)
Atenolol (Tenormin) – *also be used for acute MI*
Bisoprolol (Zebeta, Monocor)
Carvedilol (Coreg, Coreg CR) – *May also be used for heart failure, post-MI treatment*
Esmolol (Brevibloc) – *May also be used for supraventricular tachycardia*
Labetalol (Trandate)
Metoprolol (Lopressor, Toprol-XL, Betaloc) – *May also be used for acute MI, heart failure, and angina*
Nadolol (Corgard) – *May also be used to prevent rebleeding esophageal varices*
Nebivolol (Bystolic)
Propranolol (Inderal, Inderal LA, InnoPran XL) – *May also be used for supraventricular tachycardia or rapid atrial fibrillation/flutter, migraines, and to prevent rebleeding esophageal varices*

Calcium channel blockers – Dihydropyridines

Amlodipine (Norvasac)
Clevidipine (Cleviprex)
Felodipine (Plendil, Renedil)
Isradipine (DynaCirc, Dynacirc CR)
Nicardipine (Cardene, Cardene SR)
Nifedipine (Procardia, Adalat, Procardia XL, Adalat CC, Afeditab CR, Adalat XL, Adalat PA) – *May also be used for angina and pre-term labor*
Nisoldipine (Sular)

Calcium channel blockers – Non-Dihydropyridines
Diltiazem (Cardizem, Cardizem LA, Cardizem CD, Cartia XT, Dilacor XR, Diltiazem CD, Diltzac, Diltia XT, Tiazac, Taztia XT) – *May also be used to treat atrial fibrillation/flutter or angina*
Verapamil (Isoptin SR, Calan, Covera-HS, Verelan, Verelan PM) – *May also be used for supraventricular tachycardia or angina*

Diuretics – Loop
Bumetanide (Bumex, Burinex) – Mainly used for edema
Ethacrynic Acid (Edecrin) – Mainly used for edema
Furosemide (Lasix) – May also be used for edema, ascites
Rosemide (Demadex) – May also be used for edema

Diuretics - Thiazide
Chlorthalidone (Thalitone) – May also be used for kidney stones
Hydrochlorothiazide (HCTZ, Oretic, Microzide) – May also be used for edema
Indapamide (Lozol, Lozide) – May also be used for edema
Metolazone (Zaroxolyn) – Mainly used for edema

Other
Aliskiren (Tekturna, Rasilez)
Fenoldopam (Corlopam)
Hydralazine (Apresoline)
Nitroprusside (Nitropruss)
Phentolamine (Regitine, Rogitine)
Reserpine (Serpalan, Serpasil) – may also be used for severe agitation with mental disorders

Pulmonary arterial hypertension (different condition from essential hypertension)
Sildenafil (Revatio)
Tadalafil (Adcirca)

Anti-hypertensive combination drugs
Accuretic (quinapril + HCTZ)
Aldactazide (spironolactone + HCTZ)
Aldoril (methyldopa +HCTZ)
Amturnide (aliskiren + amlodipine +HCTZ)
Apresazide (hydralazine +HCTZ)
Atacand HCT (candesartan +HCTZ, Atacand Plus)
Avalide (irbesartan +HCTZ)
Azor (amlodipine + olmesartan)
Benicar HCT (olmesartan +HCTZ)
Caduet (amlodipine + atorvastatin)
Capozide (captopril + HCTZ)
Clorpres (clonidine + chlorthalidone)
Corzide (nadolol + bendroflumethiazide)

Diovan HCT (valsartan +HCTZ)
Dutoprol (metoprolol succinate +HCTZ)
Dyazide (triamterene +HCTZ)
Edarbyclor (azilsartan + chlorthalidone)
Exforge (amlodipine + valsartan)
Exforge HCT (amlodipine + valsartan +HCTZ)
Hyzaar (losartan +HCTZ)
Inderide (propranolol +HCTZ)
Inhibace Plus (cilazapril +HCTZ)
Lexxel (enalapril + felodipine)
Lopressor HCT (metroprolol +HCTZ)
Lotensin HCT (benazepril +HCTZ)
Lotrel (amlodipine + benazepril)
Maxzide (triamerene + HCTZ, Triazide)
Micardis HCT (telmisartan + HCTZ, Micardis Plus)
Minizide (prazosin + polythiazide)
Moduretic (almiloride +HCTZ, Moduret)
Monopril HCT (fosinopril +HCTZ)
Prinzide (lisinopril +HCTZ)
Tarka (trandolapril + verapamil)
Tekamlo (aliskiren + amlodipine)
Tekturna HCT (aliskirin +HCTZ)
Tenoretic (atenolol + chlorthalidone)
Teveten HCT (eprosartan +HCTZ)
Tribenzor (amlodipine + olmesartan +HCTZ)
Twynsta (amlodipine +telmisartan)
Uniretic (moexipril + HCTZ)
Caseretic (enalapril +HCTZ)
Zesoretic (lisinopril + HCTZ)
Ziac (bisoprolol +HCTZ)
